# A Dynamic 3D Human Liver Sinusoid Model for Mechanistic Interrogation of Fontan‐Associated Liver Disease

**DOI:** 10.1002/advs.202524337

**Published:** 2026-04-08

**Authors:** Sarah Rezapourdamanab, Mehdi Salar Amoli, Tanmay Mukherjee, Maryam Bagheri, Boeun Hwang, Linqi Jin, Yamini Singh, Sophia Norton, Ashay Vishwas Bongirwar, Shweta Karnik, Lakshmi Prasad Dasi, Reza Avazmohammadi, Rene Romero, Holly D. Bauser‐Heaton, Vahid Serpooshan

**Affiliations:** ^1^ Wallace H. Coulter Department of Biomedical Engineering Emory University School of Medicine and Georgia Institute of Technology Atlanta Georgia USA; ^2^ Department of Biomedical Engineering Texas A&M University College Station Texas USA; ^3^ Department of Mechanical Engineering Texas A&M University College Station Texas USA; ^4^ Division of Pediatric Gastroenterology Hepatology and Nutrition Emory University School of Medicine Atlanta Georgia USA; ^5^ Children's Healthcare of Atlanta Atlanta Georgia USA; ^6^ Department of Pediatrics Emory University School of Medicine Atlanta Georgia USA; ^7^ Sibley Heart Center At Children's Healthcare of Atlanta Atlanta Georgia USA; ^8^ Children's Heart Institute McGovern Medical School UTHealth Houston Texas USA

**Keywords:** 3D printing, biofabrication, bioprinting, disease modeling, FALD, Fontan‐associated liver disease, hepatic sinusoid

## Abstract

Fontan‐associated liver disease (FALD) is a progressive complication of Fontan circulation, driven by chronically elevated central venous pressure (CVP) and hypoxia. Current experimental models lack the fidelity to fully recapitulate the complex hemodynamic and cellular microenvironment of the liver sinusoid under Fontan physiology, limiting mechanistic insights and therapeutic discovery. We developed a perfusable, 3D bioengineered human liver sinusoid model incorporating hepatocytes and endothelial cells within a multilayered construct. The platform was cultured under tunable flow and oxygen, simulating physiological and pathological CVP. Structural and mechanical fidelity were assessed using electron microscopy and microindentation. Hemodynamic measurements by catheter and particle image velocimetry were in agreement with those predicted by computational modeling. Sinusoid analogues maintained their architecture, endothelial coverage, and hepatic viability and function over 21 days of culture. Elevated pressure and hypoxia resulted in endothelial activation (VCAM‐1, ET‐1), hepatocellular stress (HIF1α), fibronectin‐rich tissue remodeling, and altered hepatic function (albumin, bile acid, LDH, TGF‐β), consistent with early FALD pathology. Structural and molecular responses were altered with varying pressure and oxygen, confirming the platform's sensitivity to mechanical and metabolic cues. This bioengineered 3D model successfully reproduced the key early features of FALD pathophysiology, enabling controlled interrogation of pressure‐ and hypoxia‐induced liver injury.

## Graphical abstract

This study presents a perfusable 3D bioengineered liver sinusoid platform that integrates biofabrication, 3D cell culture, controlled hemodynamics, and multiparametric characterization to model Fontan‐associated liver disease. By decoupling pressure and hypoxia effects, the system reveals early mechanobiological and profibrotic responses under clinically relevant conditions, offering a scalable framework for mechanistic investigation and therapeutic screening of hepatic disorders.

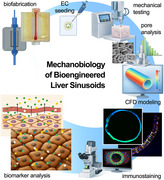

## 1 Introduction

Congenital heart diseases with single ventricle physiology represent a significant challenge in pediatric cardiology, as they involve malformations that leave either the right or left ventricle underdeveloped, effectively resulting in a single functional ventricle [1]. The Fontan operation has been the primary palliative procedure for such conditions, creating a direct pathway for systemic venous return to the pulmonary arteries without an intervening ventricle [2]. This surgical technique allows for improved arterial oxygen saturation and alleviates chronic volume overload, significantly improving survival rates into adulthood [3, 4, 5]. About 70 000 patients have undergone the Fontan procedure globally and are alive today, most of whom are under 25 years of age [6]. As of 2020, the number of individuals with Fontan circulation continues to grow, with 10‐year survival rates as high as 95% for those operated on after 2001 [7].

Despite the success of the Fontan procedure, it introduces a unique set of hemodynamic challenges. Unlike the normal cardiovascular system, the Fontan circulation lacks a subpulmonary pump, resulting in elevated central venous pressure (CVP), non‐pulsatile pulmonary blood flow, and reduced systemic output [8, 9]. This altered physiology, i.e., the *Fontan paradox*, necessitates that the CVP remains high to ensure pulmonary blood flow, in the range of 12–14 mmHg, yet low enough to avoid complications such as lymphatic stasis and edema [10]. Over time, these hemodynamic stresses, which mimic hepatic venous outflow obstruction, can lead to Fontan‐associated liver disease (FALD) [11]. FALD is characterized by chronic hepatic congestion and progressive fibrosis. Unlike other hepatic congestive diseases, resulting from backward pressure due to heart failure, FALD is compounded by the lack of pulsatile flow, leading to more diffuse liver injury with fibrosis developing silently over years, progressing to cirrhosis, and hepatocellular carcinoma [12].

FALD is driven by increased sinusoidal pressure, which is transmitted to the portal vein, exacerbating portal hypertension and resulting in liver congestion, sinusoidal dilatation, and activation of fibrogenesis [6, 11]. Pathological flow induces transcriptional upregulation and cytokine secretion in endothelial cells (ECs), that lead to thromboses [13, 14]. ECs undergo capillarization, leading to impaired oxygenation of hepatocytes as well as secretion of extracellular matrix (ECM) proteins [7]. This triggers hepatocyte necroptosis, activating hepatic stellate cells (HSCs) to become proliferative and producing ECM, further increasing stiffness [15, 16, 17, 18]. These changes result in a loss of fenestrae in ECs, distorting liver architecture, and contributing to increased intrahepatic vascular resistance and portal hypertension [19, 20, 21].

FALD is increasingly acknowledged as having distinct morphological and mechanistic differences from more conventional liver fibrotic disorders, including those caused by alcohol use or metabolic dysfunction‐related steatohepatitis [7, 22, 23]. While animal models of Fontan circulation have been developed and shown promise in investigating various pathophysiological processes (e.g., pulmonary vascular dysfunction [24, 25] and rhythmic complications [26], there are no validated animal models of FALD [27, 28, 29, 30, 31]; hence, the fundamental mechanisms underlying the FALD remain largely elusive. Animal models are also limited by high costs, low throughput, and significant anatomical and physiological differences from humans. These limitations underscore the urgent need for in vitro human‐based models of FALD that enable mechanistic studies, disease modeling, and drug testing under controlled and reproducible conditions [32]. Identifying the early and potentially driving events may provide essential understanding of FALD progression and support the development of more precise therapeutic strategies.

Current in vitro 2D and 3D models of human liver face rapid loss of function and lack tissue heterogeneity and complexity (e.g., lack of vasculature and flow), and in vivo‐like cell‐microenvironment interactions [32, 33, 34, 35]. Here, we aimed at bioengineering highly tunable, vascular, 3D models of human liver to tease out the contributions of hepatic cell‐ECM interactions and microenvironmental factors to the FALD progression. By incorporating HepG2 cells and human umbilical vein cells (HUVECs), and utilizing perfusion bioreactor systems for dynamic culture, we created a robust in vitro platform with enhanced biomimicry and throughput. This model enabled in‐depth investigation of the specific hemodynamic stresses and resultant hepatic responses characteristic of FALD.

## 2 Results and Discussion

**FIGURE 1:**
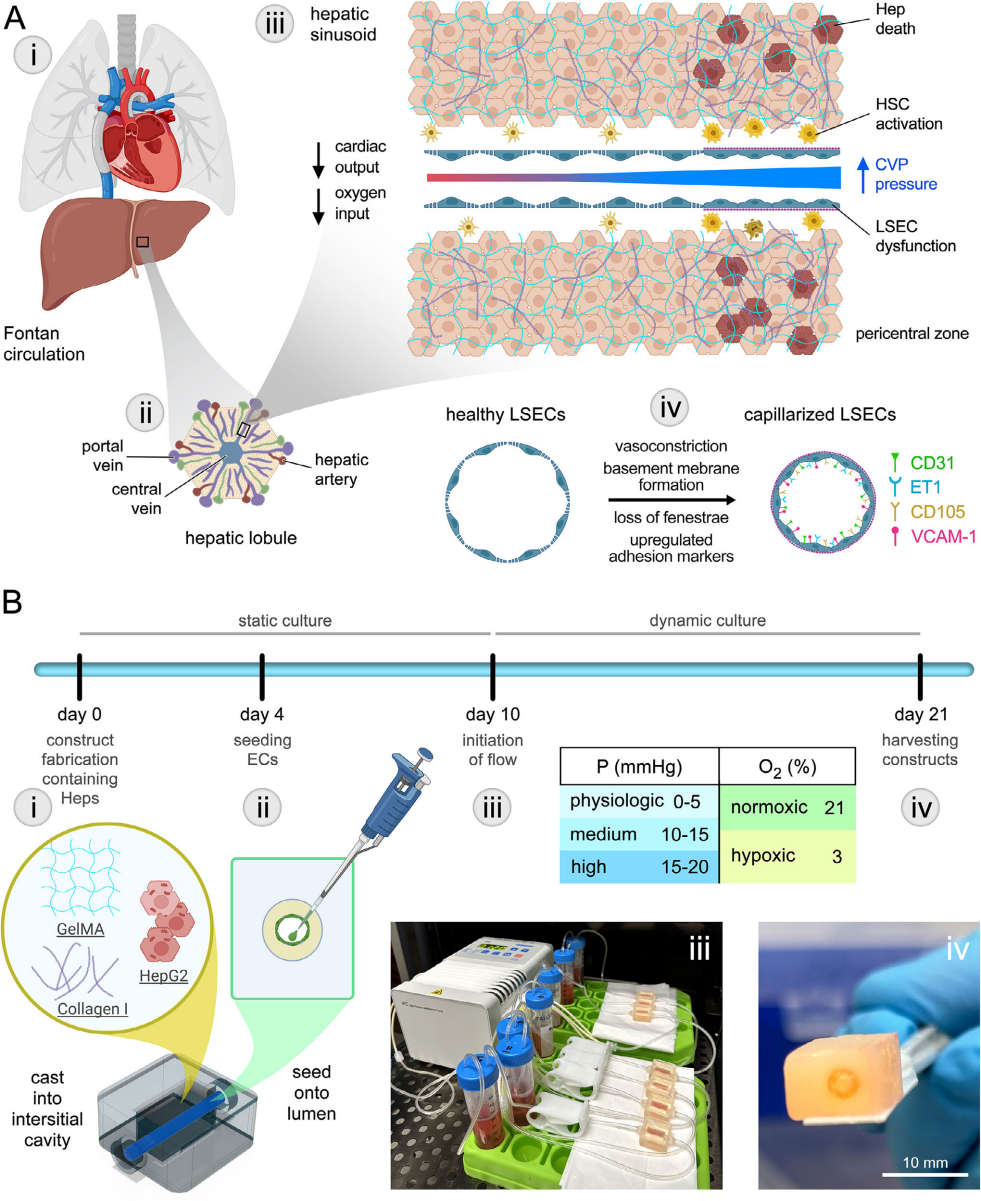
A 3D biomanufactured, perfusable human liver sinusoid model recapitulating key features of Fontan‐associated liver disease (FALD). (A) Schematic illustration of FALD pathophysiology, highlighting the Fontan circulation (i), hepatic lobule (ii), hepatic sinusoid (iii), and liver sinusoid endothelial cells (LSECs) "capillarization" (pathological transformation) (iv). The elevated central venous pressure (CVP), hypoxia, hepatic fibrosis, hepatocyte (Hep) death, LSEC dysfunction, and hepatic stellate cell (HSC) activation are illustrated. (B) Experimental timeline and workflow to manufacture 3D FALD models, including Day 0: casting 3D constructs with human hepatocytes (HepG2 cells) encapsulated in a hydrogel (i), Day 4: manual seeding of the channel lumen with ECs (ii), followed by Day 10: dynamic perfusion under controlled flow and oxygen conditions (the table) (iii), and finally Day 21: harvesting the constructs at the end of 3‐week culture (iv).

Current understanding of FALD remains limited, with few experimental systems capable of modeling the complex interplay between hemodynamic forces and hepatic cellular responses. Although clinical studies have highlighted the multifactorial nature of FALD, including the role of chronically elevated CVP and impaired oxygenation in driving hepatic congestion and fibrosis, the direct mechanistic links between Fontan physiology and progressive liver injury remain elusive [6, 8, 13, 36]. There is a lack of experimental models that can simultaneously capture these hemodynamic and oxygenation variables in a biomimetic hepatic microenvironment [7, 27]. To address this gap, we developed a 3D biomanufactured, perfusable human liver sinusoid model that integrates perfusion dynamics and oxygen control to simulate post‐Fontan physiology (Figure 1). In designing this model, we aimed at recapitulating the Fontan circulation within the human hepatic tissue structure (sinusoids), i.e., the elevated CVP, hypoxia, hepatic fibrosis, hepatocyte death, and liver sinusoidal EC (LSEC) dysfunction (Figure 1A).

Fabricated model integrated a multilayered structure composed of hydrogel‐encapsulated hepatocytes (HepG2 cells), forming the bulk hepatic tissue, and ECs lining onto the central channel lumen (Figure 1B,i‐ii). Following an initial 10‐day static culture, constructs were cultured under dynamic flow for 11 days, simulating physiological versus pathological CVP, and normoxic versus hypoxic environments (Figure 1B,iii‐iv). The first 10 days of static culture were necessary to allow stable hepatocyte‐ECM integration and complete endothelial barrier formation prior to perfusion. Preliminary assessments demonstrated that shorter static culture periods resulted in incomplete endothelial coverage and reduced lumen integrity under flow.

**FIGURE 2:**
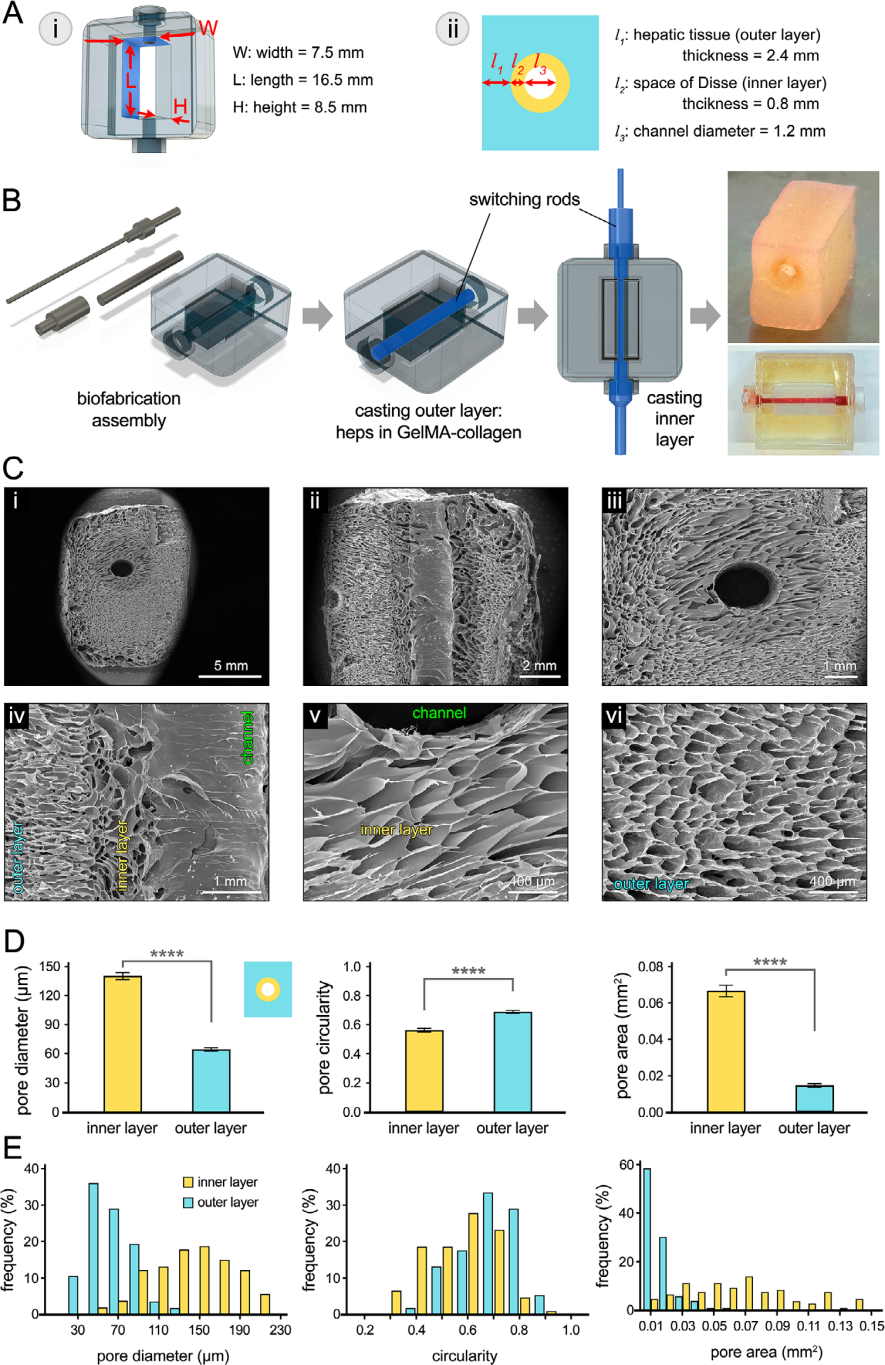
Characterization of the 3D engineered models of hepatic sinusoids. (A) Schematic representation of the housing design (i) and the cross‐sectional view of sinusoid construct design (ii), illustrating the dimensions of various components. (B) Experimental workflow to fabricate hepatic sinusoid constructs. From left to right, different steps of the biofabrication process are shown. First, a larger‐diameter plastic rod is inserted into the channel space, allowing the cast the outer layer hydrogel containing hepatocytes (Heps). After crosslinking the outer layer, the rod is replaced with a smaller‐diameter one, leaving an empty space around the central channel for casting the inner layer. After crosslinking the inner layer, the smaller rod will be removed as well, resulting in formation of a 2‐layer soft construct with a patent channel allowing dynamic culture. (C) Scanning electron microscopy (SEM) images of the biofabricated sinusoid constructs at varying magnifications, presenting cross‐sectional (i, iii, v, vi) and longitudinal (ii, iv) views. The outer layer (hepatic tissue), inner layer (space of Disse), and the channel are highlighted. (C,D) Quantitative analysis of pore size and distribution based on the SEM images, demonstrating pore diameter, circularity, and surface area for the inner and outer layers of liver constructs. Data are presented as mean ± SD (*n* = 3 constructs per group). Statistical significance was determined using one‐way ANOVA followed by Tukey's multiple comparison test. ****: *p* < 0.0001.

The dimensions of the FALD constructs were optimized to balance physiological relevance with experimental feasibility (Figure 2A). This design minimized material and cell usage while ensuring compatibility with perfusion, reproducibility, and scalability. The 3D scaffold architecture included a central perfusable lumen, mimicking the sinusoid, an inner layer mimicking the space of Disse, and an outer layer populated with hepatocytes, to resemble the liver lobule tissue (Figure 2B,C). It is important to note that while the inner layer was designed to accommodate HSCs in future studies, it was left acellular in the present work to ensure feasibility (Figure 2B,C). This layered configuration reflects the anatomical organization of human liver sinusoids by incorporating the main structural features and relevant cell compartments for modeling FALD, while remaining feasible for routine fabrication, long‐term culture, and experimental manipulation [34, 35, 37].

SEM imaging of the 3D liver sinusoid constructs revealed a highly porous architecture and well‐preserved central lumen geometry suitable for cell growth and media exchange (Figure 2C; Figure S1). The bilayer composition of the constructs was clearly distinguishable in SEM images, with each layer demonstrating distinct pore structure and surface topography, attributable to differences in the bioink formulations used for the inner and outer layers (Figure 2C). The use of GelMA‐based hybrid hydrogels has been validated in prior studies for providing mechanically tunable, cytocompatible environments conducive to endothelial and hepatic function [38, 39, 40]. These results confirm the ability of the employed multi‐material biofabrication strategy in creating precise spatial heterogeneity within the 3D scaffolds.

Quantitative pore analysis of SEM images revealed significantly (*p* < 0.0001) larger average pore diameter and surface area in the inner layer compared to the outer layer, confirming the intentional material‐driven design of the scaffold microstructure (Figure 2D,E). The remarkable differences in pore structure of the two layers could be attributed to the distinct bioink compositions used to create these layers, i.e., 5% GelMA and 2.7 mg/mL collagen for the outer layer versus 10% GelMA for the inner layer. While lowering GelMA concentration was necessary to maintain physiologic liver stiffness (<5 kPa) in the outer layer, reducing polymer content alone decreases crosslink density, which can promote hydrogel swelling and result in enlarged pore size. To counteract this effect while preserving mechanical softness, collagen type I was incorporated into the outer layer. As a native ECM component of liver parenchyma, collagen undergoes thermal fibrillogenesis prior to GelMA photopolymerization, forming a fibrillar network that interpenetrates the subsequently crosslinked GelMA matrix. This sequential crosslinking approach creates a structurally organized, interpenetrating network that restricts excessive pore expansion without substantially increasing bulk stiffness. Consequently, the outer layer achieves controlled porosity representative of hepatic tissue while maintaining physiologic compliance and mechanical stability under perfusion.

Photocrosslinking of the inner layer, cast after the outer layer, may have been hindered by the barrier effect of the outer layer, which limited blue light penetration into the hydrogel and consequently reduced pore density. The increased porosity in inner layer is advantageous for facilitating endothelial adhesion, proliferation, and mechanosensing under flow, in analogy to the native endothelium function in the sinusoids [38, 40, 41]. In contrast, the outer layer, which contains encapsulated HepG2 cells, presented a denser and more compact fibrillar structure with smaller pores. This reduced porosity is favorable for promoting hepatocyte‐ECM interactions, helping to maintain cell morphology and function within a confined and physiologically relevant microenvironment [38, 40, 41]. Of note, the inner layer also showed a more elongated pore shape with significantly (*p* < 0.0001) reduced circularity compared to the pores in outer layer (Figure 2C–E), which could also be related to the different bioink compositions and crosslinking conditions.

**FIGURE 3:**
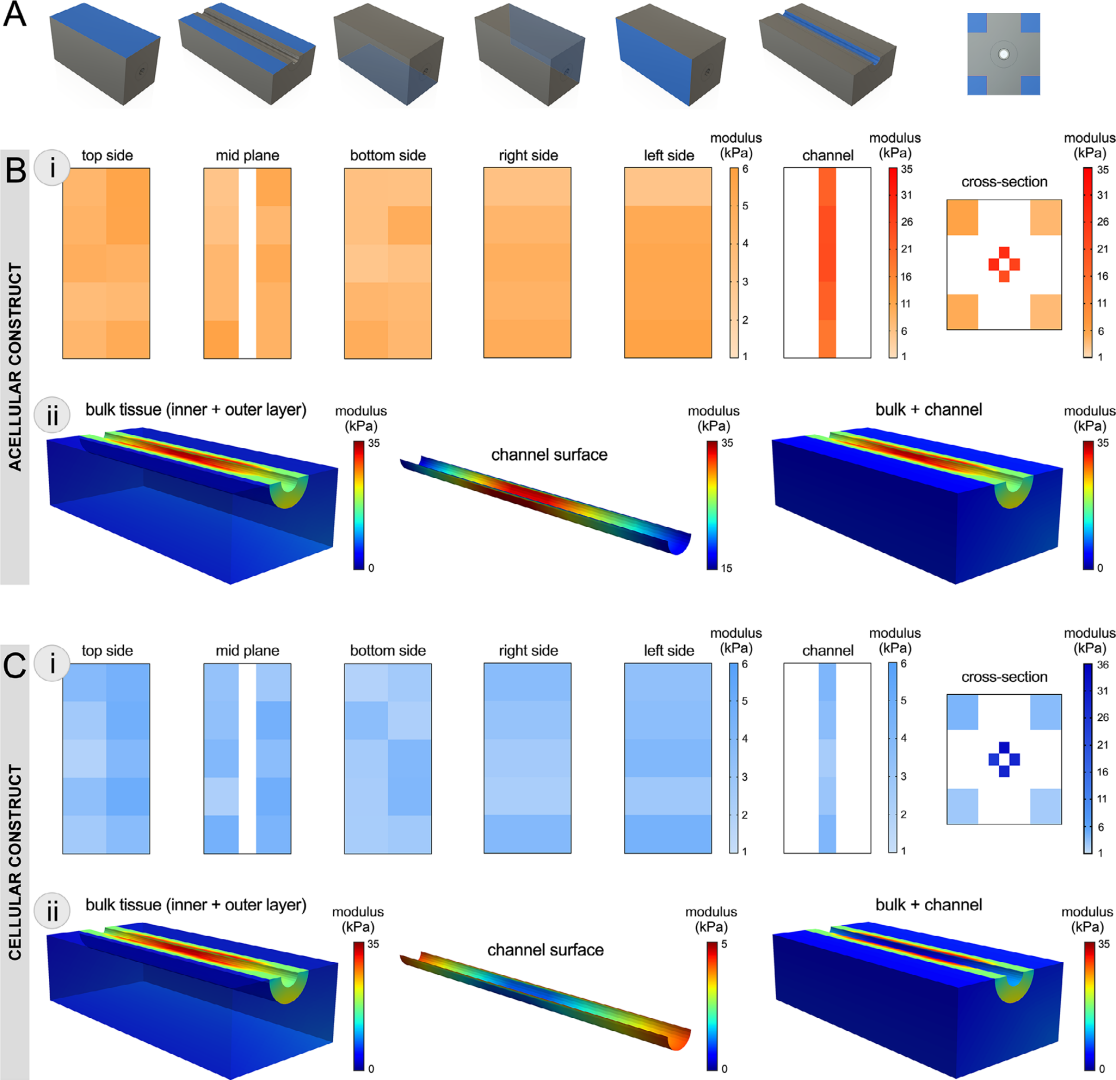
Micromechanical characterization of 3D biomanufactured, perfusable human liver sinusoid constructs. (A) Schematic illustration of various surfaces used to probe elastic modulus within the 3D constructs via an array of microindentation tests. (B,C) Microindentation tests were conducted on the acellular (B) and cellular (3 weeks of coculture) (C) sinusoid constructs, on the top, mid plane, bottom, right, and left sides, the lumen surface, and the perpendicular cross‐section (i). These 2D (i) and 3D (ii) heatmaps demonstrate the distribution of elastic modulus within each group of constructs (*n* = 4 per group). Data are presented as mean ± SD.

Micromechanical analysis of constructs confirmed the fabrication of a compliant, biomimetic multi‐material matrix that closely replicated the mechanical microenvironment of hepatic parenchyma, averaging under 6 kPa (Figure 3) [42]. Comparison of acellular versus cellular groups showed that the constructs, while soft, preserved their structural integrity over 21 days of dynamic culture, highlighting the mechanical stability and design tunability (Figure 3B,C). Numerical extrapolation of elastic modulus measurements within the 3D geometries further demonstrated a relatively uniform distribution of stiffness (0–6 kPa) within the outer layer (i.e., the hepatic tissue) (Figure 3B,C). The inner layer exhibited higher elastic moduli (∼30–35 kPa), consistent with its role in structural reinforcement in this study to establish the endothelium. The increased stiffness observed in the inner layer can be attributed to its higher GelMA concentration (10%), relative to the 5% used for the outer layer. The incorporation of cells did not significantly affect the properties of the haptic (outer) layer; however, lumen seeding (coating) with HUVECs caused a significant, and anticipated, decrease in the measured modulus. These findings underscore the suitability of engineered platform for modeling soft tissue microenvironments like the human liver, where matrix compliance plays a key role in cell signaling, differentiation, and fibrosis/disease progression [23, 35]. In future phases of the study, with the incorporation of HSCs in this layer, further modulation of mechanical properties for this layer might be necessary [43].

To replicate FALD conditions in our engineered hepatic sinusoid constructs, we sought to mimic the elevated CVP characteristic of Fontan circulation. Establishing this pathophysiologically relevant pressure profile was essential to evaluate whether the model could accurately reproduce the hemodynamic environment of the disease. We conducted precise intraluminal pressure measurements using a Volcano pressure wire system in a catheterization laboratory setting (Figure S2). Pressure was measured across a range of flow rates (0.12–0.50 mL/min), with and without downstream resistance to simulate venous outflow obstruction (Table 1; Figure S2).

**TABLE 1:** List of flow rates employed and pressure values measured using a Volcano pressure wire system in the middle of the hepatic vein‐like channel.

flow rate (mL/min)	normalized pressure in mid‐channel (mmHg)	
	without resistance	with resistance
0	0	0
0.12	0‐5	0‐5
0.15	0‐7	0‐6
0.2	2‐8	2‐9
0.3	2‐9	6‐13
0.4	2‐10	10‐19
0.5	3‐10	11‐21

The flow‐pressure relationship revealed a nearly linear increase in intraluminal pressure as perfusion rate increased, with significantly elevated pressures observed in the presence of outflow resistance (Figure S2C). At 0.5 mL/min, constructs without the resistance maintained pressures below 5 mmHg, within the physiological range for healthy CVP (Table 1; Figure S2C). In contrast, with resistance applied, pressure rose steeply to over 15 mmHg, effectively modeling the elevated CVP characteristic of Fontan physiology and FALD (Table 1; Figure S2C) [23, 35]. This ability to tune intraluminal pressure by adjusting flow rate and resistance confirmed the mechanical responsiveness of the platform and its suitability for simulating both normal and pathological hemodynamic environments. Notably, the pressure range achieved in this setup (0.5–21 mmHg) encompasses the physiological to pathophysiological CVP spectrum, enabling the study of endothelial and hepatic responses under controlled flow conditions [44, 45, 46]. Such control is critical for investigating pressure‐induced endothelial dysfunction, sinusoidal remodeling, and fibrogenic signaling in a FALD‐specific context [47, 48].

**FIGURE 4:**
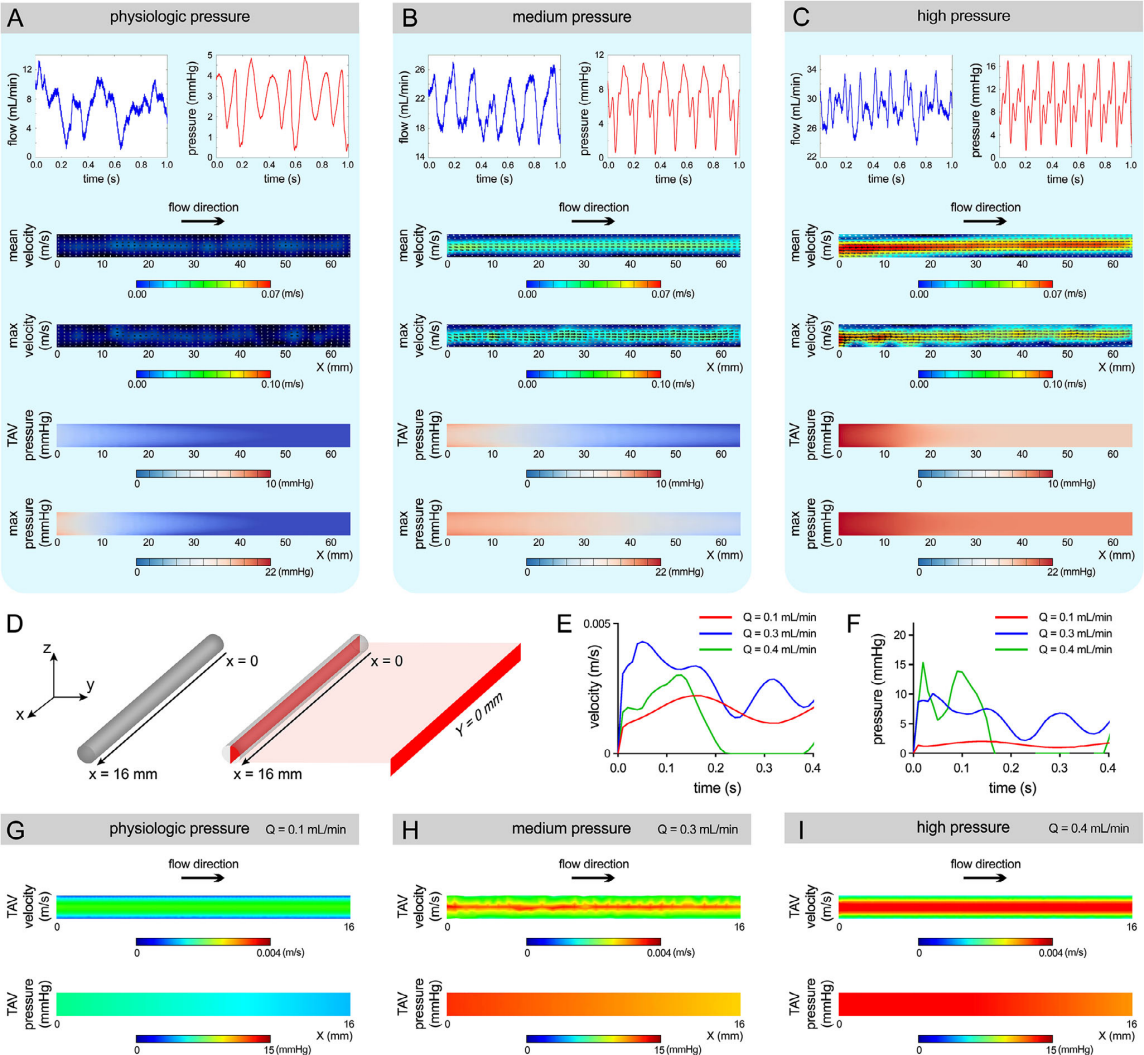
Characterization of flow hemodynamics within perfused in vitro models of hepatic sinusoid. (A–C) The use of particle image velocimetry (PIV) to measure flow parameters within the 3D geometry at a 4X scale. Flow rate and pressure time‐series plots (top row) under three inlet flow conditions (7.7, 19.2, and 25.6 mL/min) with outlet resistance, representing physiological (A), medium (B), and high (C) central venous pressure (CVP) conditions, respectively. PIV‐derived spatial maps of mean and maximum velocity magnitudes across the channel under each flow condition are presented (rows 2‐3). Further, pressure distribution showing time‐averaged (TAV) and maximum intraluminal pressure along the direction of flow are presented (rows 4‐5). (D–I) Computational fluid dynamics (CFD) modeling of flow within the 3D models at 1X scale. (D) 3D rendering of the CFD simulation domain geometry used to replicate the sinusoid construct. (E) Velocity magnitude profiles obtained from CFD simulations across three input flow rates (0.1, 0.3, and 0.4 mL/min), confirming a progressive increase in flow velocity at higher perfusion rate. (F) Simulated pressure profiles showing spatial and temporal changes across the construct length. (G–I) Axial maps of velocity and pressure distributions at low (physiologic), medium, and high flow/pressure settings.

To validate and visualize flow behavior within the sinusoid constructs under varying disease‐mimicking conditions, we employed laser PIV technique using a 4X scaled‐up transparent model of the perfusion channel geometry. This allowed for high‐resolution measurement of flow velocity and pressure distribution under three distinct conditions: physiological (baseline), moderate, and high pressure conditions, corresponding to increasing levels of simulated CVP (Figure 4) [6, 45, 49]. Flow rate and pressure time‐series plots captured cyclical fluctuations inherent to the pulsatile input system. As expected, pressure amplitude and baseline values increased with higher flow rates and outflow resistance (Figure 4A–C). These pressure traces confirmed that the system successfully recapitulated physiological (0–5 mmHg) and pathological (10–20 mmHg) CVP, creating a platform suitable for modeling the hemodynamic range relevant to Fontan circulation and FALD [46, 50].

PIV flow field reconstructions provided further insight into spatial and temporal hemodynamics. Cross‐sectional maps of mean and maximum velocity magnitudes across the channels were measured under each pressure condition (Figure 4A–C, rows 2‐3) [46, 51]. As flow rate increased from baseline to high‐pressure settings, both peak and average velocities increased proportionally, with notable changes in velocity gradient steepness at the channel walls. These shear gradients are particularly relevant for studying endothelial mechanotransduction, as previous studies have shown that elevated shear can trigger endothelial inflammation and loss of fenestration [49, 52, 53, 54].

Corresponding pressure from PIV distribution maps were computed and revealed a progressive elevation in both time‐averaged and peak intraluminal pressure along the direction of flow (Figure 4A–C. rows 4‐5) [55]. At the high‐pressure condition, spatial pressure gradients became more pronounced, reflecting the downstream resistance imposed via the introduced tube clamping. The system reproduced realistic flow profiles and pressure patterns observed in vivo. Collectively, these PIV results confirmed that the bioreactor and perfusion system successfully generated a physiologically tunable flow environment [54, 56].

To complement experimental PIV measurements and extend hemodynamic analysis to scales not easily accessible via imaging, computational fluid dynamics (CFD) simulations were performed at 1X scale (i.e., the scale of bioprinted constructs) [57, 58]. Pressure and Reynolds number were kept constant at the inlet to reflect the physiological intraluminal pressure and low Reynolds flow typical of hepatic microcirculation [59]. The fluid was modeled as incompressible, Newtonian, and steady state, with boundary conditions adjusted to match the perfusion flow rate used in experiments. These simulations allowed for the characterization of velocity fields and pressure gradients across physiological and pathophysiological perfusion settings relevant to FALD [60]. CFD results showed agreement with experimental PIV velocity profiles, validating model assumptions and confirming that the flow remained laminar under all test conditions (Figure 4D–I). Simulations revealed clear increases in velocity magnitude and pressure drop across the channel length with elevated inlet flow rates, corresponding to medium and high CVP‐like conditions [58, 60].

Velocity plots obtained from CFD modeling predicted increased central flow velocities and evolving flow profiles with elevated perfusion (Figure 4E). Although the simulations were steady state, the spatial velocity gradients aligned with the pressure dynamics observed in PIV, suggesting increasing mechanical stress within the construct. Wall shear stress was not directly computed or measured; however, the velocity distribution near the boundaries may imply flow‐induced stress patterns [57]. The pressure curves showed consistent spatial trends, with increasing mean pressure values as outflow resistance was applied (Figure 4F), reproducing the pressure amplitudes and gradients observed experimentally in PIV (Figure 4A–C) [58, 60].

CFD also provided axial pressure maps, which in high‐pressure conditions induced steeper axial pressure gradients, hallmarks of the pathophysiologic microenvironment experienced in the post‐Fontan liver (Figure 4G–I). These gradients are especially important in regions mimicking the sinusoidal inlet and pericentral zones, where ECs are most sensitive to mechanical stress [61, 62]. Furthermore, pressure drop analysis confirmed a progressive increase in hydraulic resistance across the construct with each stepwise increase in flow rate and resistance, supporting the concept that the engineered platform can faithfully replicate the hemodynamic burden characteristic of FALD [60, 62].

Taken together, the CFD simulations reinforce experimental findings and demonstrate the precision and scalability of the model. By enabling full spatiotemporal resolution of flow and pressure within perfusable constructs, CFD provides a robust computational tool for tuning model parameters and predicting cellular response regions. Importantly, CFD modeling bridges the gap between idealized experimental conditions and complex in vivo realities, enhancing the platform's translational potential for disease modeling and therapeutic testing [57, 60, 62].

**FIGURE 5:**
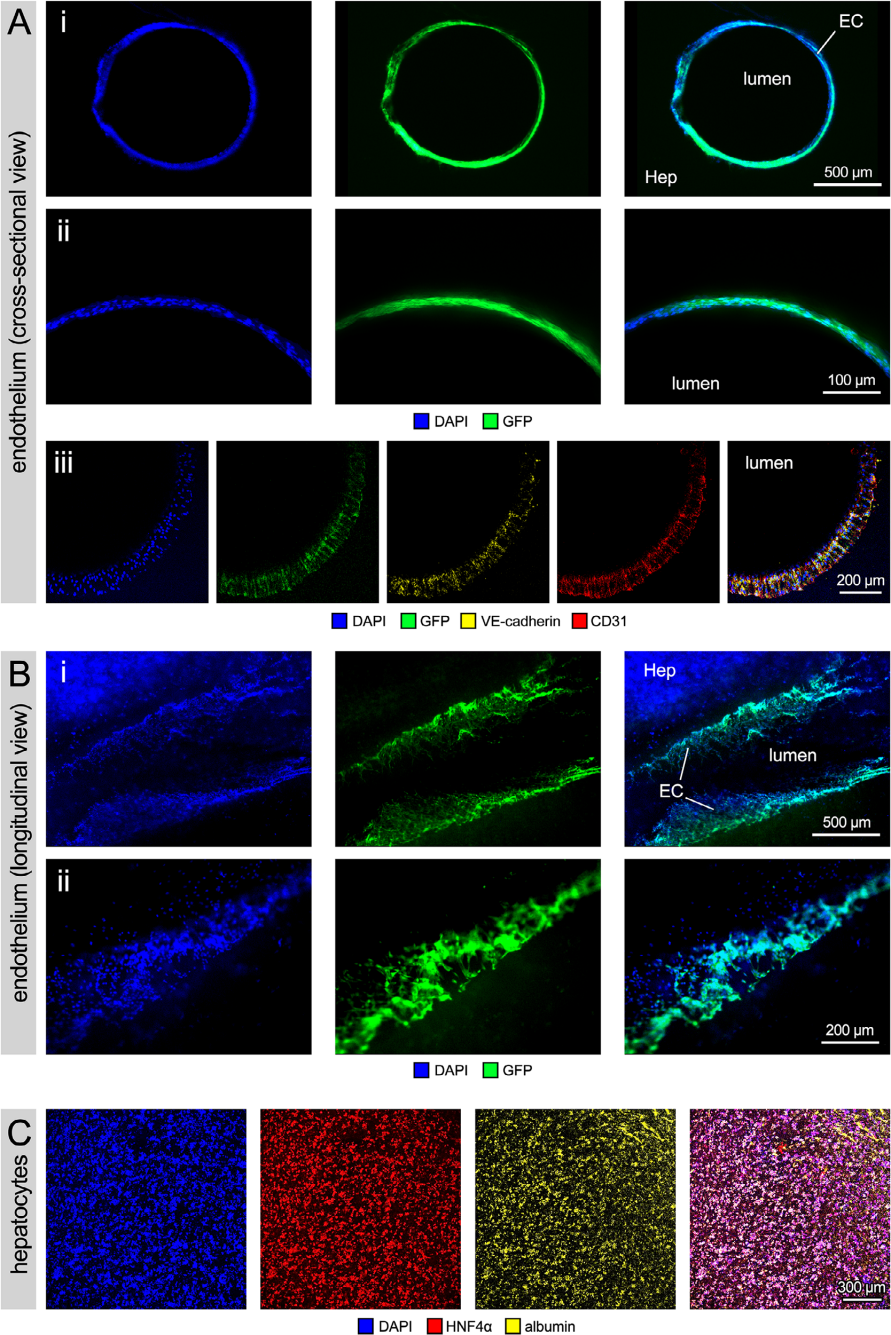
Immunostaining analysis of endothelial (EC) and hepatic (Hep) cell behavior in the 3D perfused sinusoid models. (A,B) Immunostaining images of the stablished endothelium, consisting of human umbilical vein ECs (HUVECs), on the luminal surface of channel. Cross‐sectional (A, i‐iii) and longitudinal (B, i‐ii) views confirm robust GFP‐positive endothelial coverage along the lumen, with DAPI counterstaining for nuclei. Immunostaining for VE‐cadherin and CD31 (A, iii) demonstrates mature EC phenotype with tight junctional organization and membrane localization. (C) Human Hep (HepG2) cells, encapsulated in the outer layer of 3D constructs, maintain hepatic identity under dynamic 3D culture, as evidenced by strong nuclear HNF4α and cytoplasmic albumin expression. All images were obtained after 14 days of 3D culture, including 4 days under dynamic perfusion.

The viability and phenotypic stability of cells incorporated within the 3D structures are critical for establishing a reliable in vitro model of the liver sinusoid [33, 63]. To evaluate cell localization, morphology, and marker expression under physiological flow conditions, we performed immunostaining on liver sinusoid constructs harvested on day 14, following 4 days of dynamic culture (Figure 5). This intermediate time point was selected to validate structural integrity and baseline cellular phenotype under controlled perfusion conditions. Longer‐term responses and stress‐dependent remodeling were evaluated at later stages of this work. A series of 2D culture assays for both HUVEC and HepG2 cells were used to determine the optimal culture media composition for the 3D coculture experiments (Figure S3A,B). Cross‐sectional (Figure 5A) and longitudinal (Figure 5B) imaging of the lumen revealed a robust, continuous, and uniformly distributed layer of GFP‐labeled HUVECs, indicating successful seeding, retention, and channel coverage under dynamic flow. Co‐staining for endothelial markers VE‐cadherin and CD31 demonstrated strong junctional and membrane localization, consistent with stable endothelial cell‐cell contacts and a mature endothelium (Figure 5A,iii). This tight junctional organization is essential to mimic sinusoidal permeability and barrier function in vitro [52, 64]. The preserved circular morphology of the lumen and uniform marker expression along the circumference highlight the structural and functional integration of HUVECs within the perfused system. This architecture mimics the fenestrated and low‐shear environment of native liver sinusoids, which is essential for accurate modeling of pressure‐ and flow‐ induced endothelial responses [65, 66].

In the outer hepatocyte‐rich layer, HepG2 cells remained viable and expressed key hepatic markers, including HNF4α (nuclear) and albumin (cytoplasmic), indicating active hepatic function (Figure 5C). Evidence of progressive increases in nuclear density and time‐dependent tissue compaction over the 21‐day culture period was observed by DAPI imaging (Figure S3D), supporting the long‐term viability and scalability of hepatocyte encapsulation in a soft 3D hydrogel [34, 67]. The results from HepG2 monocultures in both 2D and 3D systems demonstrate time‐dependent growth and compaction during extended culture (Figure S3D). These findings demonstrate that encapsulated HepG2 cells maintain a healthy phenotype and synthetic capacity under prolonged 3D culture with dynamic perfusion, supporting the platform's utility for disease modeling. The simultaneous preservation of endothelial and parenchymal features under shared hemodynamic and oxygenation settings strengthens the physiological relevance of the system. Together, these immunostaining results confirm the successful establishment of a multicellular liver sinusoid analogue with adequate structural fidelity and tissue‐specific marker expression.

**FIGURE 6:**
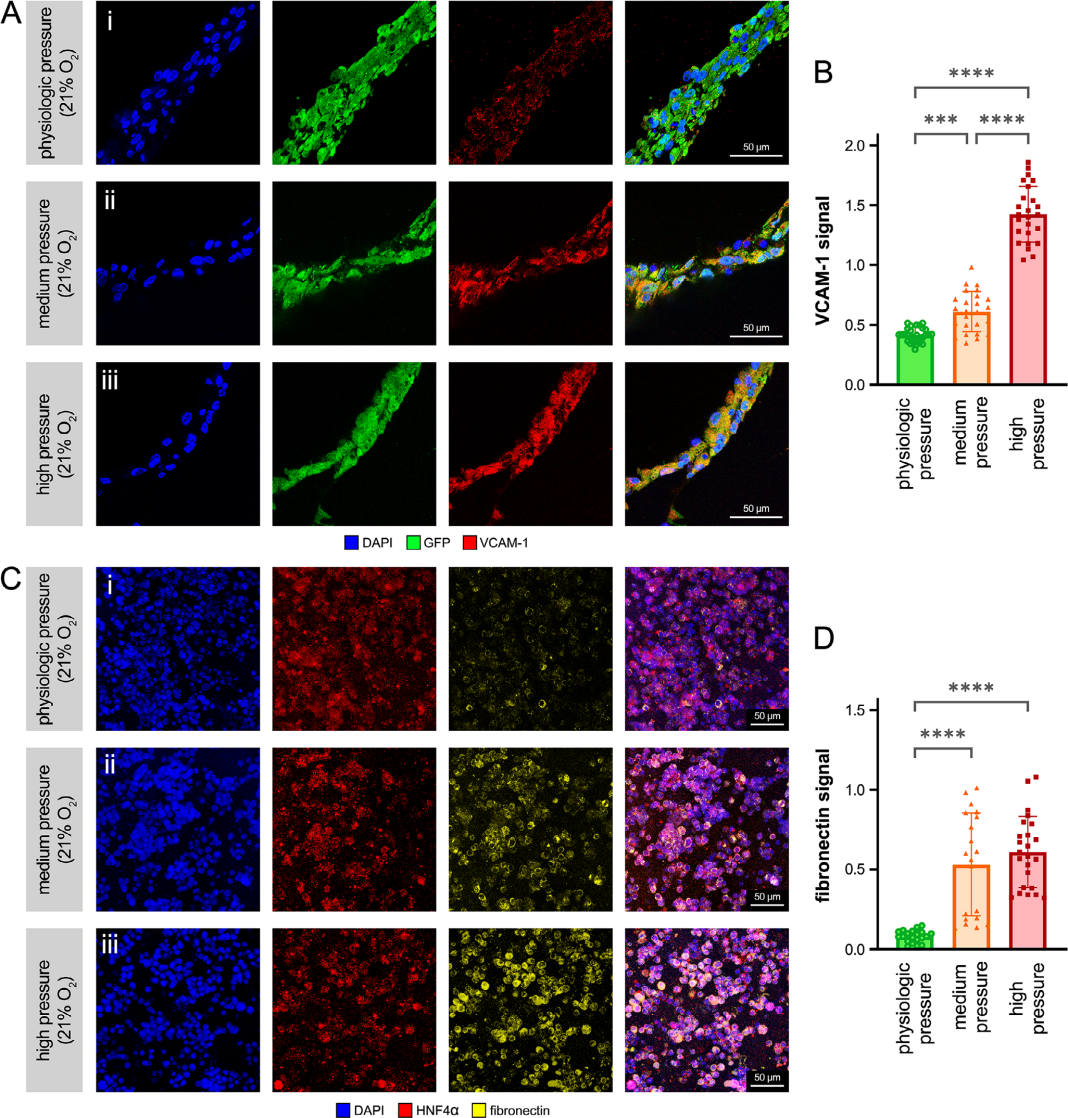
Pressure‐dependent endothelial activation and hepatic matrix remodeling in perfused liver sinusoid constructs under normoxic (21% O_2_) conditions. (A) Immunostaining of the endothelial‐lined lumen in 3D sinusoid constructs, showing increased VCAM‐1 expression with increasing intraluminal pressure (physiologic ∼0–5 mmHg, medium ∼10–15 mmHg, and high pressure ∼15–20 mmHg). GFP (green) and DAPI (blue) staining confirm monolayer endothelial coverage, while VCAM‐1 (red) signal increases notably under high‐pressure perfusion. (B) Quantification of VCAM‐1 expression (normalized by DAPI signal) for various perfusion pressure groups (*n* = 3 per group). (C) HepG2 cells in the outer layer of constructs were stained for HNF4α (red) and fibronectin deposition (yellow) across all pressure conditions. (D) Quantification of fibronectin expression (normalized by DAPI signal) for all pressure groups (*n* = 3 per group). Data are presented as mean ± SD. Statistical significance was determined using one‐way ANOVA followed by Tukey's multiple comparison test. ***: *p* < 0.001; ****: *p* < 0.0001.

To evaluate the cellular responses to hemodynamic stress relevant to Fontan physiology, we examined the effect of physiologic (∼0–5 mmHg), medium (∼10–15 mmHg), and high (∼15–20 mmHg) pressure conditions on endothelial and hepatic compartments under normoxic (21% O_2_) dynamic culture. Immunostaining on day 21 (11 days of perfusion culture under pressure) revealed distinct pressure‐dependent alterations in cellular phenotype and matrix remodeling (Figure 6).

Immunostaining of the central lumen demonstrated a marked upregulation of vascular cell adhesion molecule 1 (VCAM‐1) with increasing pressure, while GFP and DAPI co‐localization confirmed endothelial monolayer continuity (Figure 6A). Quantitative fluorescence intensity analysis (Figure 6B) showed significantly (*p* < 0.0001) elevated VCAM‐1 expression under high‐pressure condition compared to both medium and physiologic groups. At physiologic pressure, VCAM‐1 expression was minimal, consistent with a quiescent endothelial phenotype [68]. Under medium‐pressure condition, VCAM‐1 elevation was observed along the lumen (*p* < 0.001), suggesting early signs of endothelial activation. Notably, high‐pressure condition induced a robust VCAM‐1 expression pattern, indicative of strong proinflammatory signaling and loss of endothelial homeostasis [69]. These findings are consistent with the mechanosensitive nature of LSECs, which are known to shift from a tolerogenic to proinflammatory state under elevated shear and pressure, contributing to immune cell recruitment and fibrotic progression in FALD and other hepatic pathologies [64, 66, 70, 71].

Further immunostaining of endothelium showed that HUVECs expressed CD105, an established marker of endothelial remodeling and angiogenic response [72, 73], as well as fibronectin deposition particularly under high‐pressure flow demonstrating matrix remodeling in response to mechanical stress [74] (Figure S4A,B). These observations align with previous reports demonstrating EC mechanosensing leading to ECM remodeling and inflammatory activation in liver fibrotic diseases [64].

HepG2 cells in the outer layer exhibited pressure‐dependent increase in extracellular fibronectin deposition and sustained expression of HNF4α, a hepatic transcription factor (Figure 6C). Under physiologic pressure, fibronectin presence was sparse and pericellular. In contrast, exposure to medium pressure led to an evident upregulation in extracellular fibronectin intensity and distribution. This effect was further amplified under high‐pressure flow, which exhibited widespread matrix remodeling surrounding the encapsulated hepatocytes (Figure 6C; Figure S4D). Quantitative analysis of fibronectin signal confirmed statistically significant (*p* < 0.0001) increases in the medium‐ and high‐ pressure groups compared to the physiological baseline (Figure 6D). This suggests that even in normoxic conditions, mechanical cues alone can trigger parenchymal matrix remodeling and potential pre‐fibrotic responses [75, 76, 77]. This mechanosensitive behavior of hepatocytes and their surrounding ECM mirrors observations in cirrhotic and congestive hepatopathies, where mechanical stress precedes the fibrotic cascade initiation [78, 79].

**FIGURE 7:**
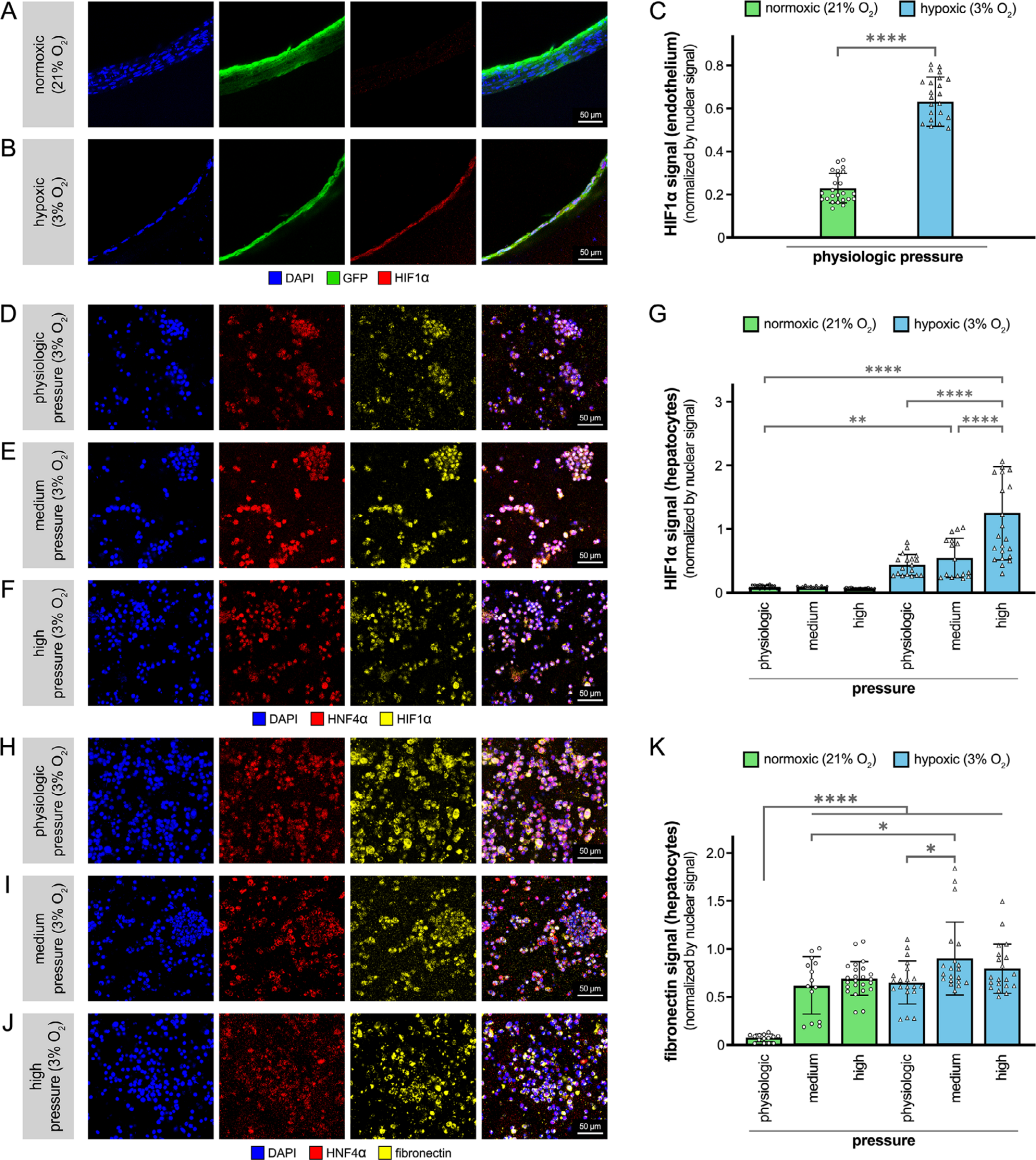
Hypoxia induces HIF1α expression and fibronectin deposition in hepatocyte and endothelial compartments in 3D perfused models of sinusoids. (A,B) Immunostaining of human umbilical vein endothelial cells (HUVECs) in the central lumen of constructs, cultured under physiological pressure, in normoxic (A) and hypoxic (B) conditions. Constructs exposed to 3% O_2_ showed strong nuclear HIF1α staining in GFP‐labeled HUVECs (green), compared to the markedly reduced signal in the normoxia group. (C) Quantification of signal confirms significantly elevated endothelial HIF1α expression under hypoxia (p < 0.0001). (D–F) Immunostaining analysis of human hepatocyte (HepG2) cells encapsulated in the outer layer of 3D constructs, cultured in hypoxic (3% O_2_) conditions, under physiologic, medium, and high pressures. These results are compared against the immunostaining data presented for the normoxic (21% O_2_) culture. These images show strong nuclear localization of HIF1α (yellow) in hypoxia groups, indicating oxygen‐sensitive stress activation. (G) Quantification of nuclear HIF1α signal reveals significant upregulation in all hypoxic groups compared to normoxic control (*p* < 0.01). (H–J) Immunostaining demonstrates fibronectin (yellow) deposition in the extracellular matrix (ECM) across hypoxic conditions, independent of pressure. Nuclei are counterstained with DAPI (blue), and HNF4α (red) marks the hepatocyte phenotype. These results are compared against the immunostaining data presented for the normoxic culture (Figure 6). (K) Quantification of fibronectin signal. A sample size of *n* = 3 per group was used for all quantification analyses. Data are presented as mean ± SD. Statistical comparisons were performed using two‐way ANOVA with Tukey's multiple comparison post‐hoc test. **: *p* < 0.01 and ****: *p* < 0.0001.

To investigate the effects of oxygen deprivation on endothelial and hepatic behavior within the 3D sinusoid‐mimicking constructs, we cultured the models under controlled hypoxic (3% O_2_) conditions (Figure 7) and compared with those cultured in normoxic (21% O_2_) conditions (Figure 6). We first examined the impact of oxygen deprivation on sinusoidal endothelium at physiologic pressure. Constructs cultured in hypoxia exhibited significantly (*p* < 0.0001) increased HIF1α expression in ECs nuclei, while normoxic controls showed negligible signal (Figure 7A–C). Thus, even in the absence of elevated pressure, oxygen deprivation alone activated hypoxia‐response pathways in the endothelium. These results supported previous findings that LSECs respond rapidly to oxygen deprivation, even in the absence of elevated pressure, by activating hypoxia‐responsive pathways [80, 81, 82, 83].

Immunostaining of HepG2 cells in the outer layer revealed a pronounced increase in HIF1α expression in constructs cultured under hypoxic (3% O_2_) conditions, and this effect was further amplified at higher pressures (Figure 7D–F). Nuclear localization of HIF1α, particularly in the high‐pressure group, indicates that hypoxia is the primary driver of cellular stress, with elevated pressure serving as an additional amplifier [84, 85, 86]. Quantification confirmed significant upregulation of HIF1α across all hypoxic groups compared to the normoxic controls (Figure 7F), consistent with prior studies showing that HIF1α is a key regulator of hepatic response to oxygen deprivation and a precursor to metabolic reprogramming and fibrogenesis [87, 88, 89, 90].

Fibronectin staining in the hepatic tissues showed a substantial increase in ECM deposition under hypoxia in all pressure conditions (Figure 7G–I) compared to normoxic physiological pressure control (Figure 6C,D). This finding suggests that oxygen deprivation alone was sufficient to induce matrix remodeling, independent of perfusion pressure. This ECM response is commonly seen in early‐stage fibrotic remodeling and aligns with prior observations in congestive hepatopathies and cirrhosis, where chronic hypoxia promotes fibronectin deposition, hepatocyte dedifferentiation, and capillarization of sinusoids [91, 92, 93]. Together, the immunostaining results emphasize that oxygen tension is a dominant regulator of both endothelial and hepatic responses in these in vitro models. While elevated pressure adds to the cellular stress, hypoxia independently drives HIF1α activation, ECM remodeling, and phenotypic adaptation, validating the model's utility in studying oxygen‐sensitive liver pathologies such as FALD.

**FIGURE 8:**
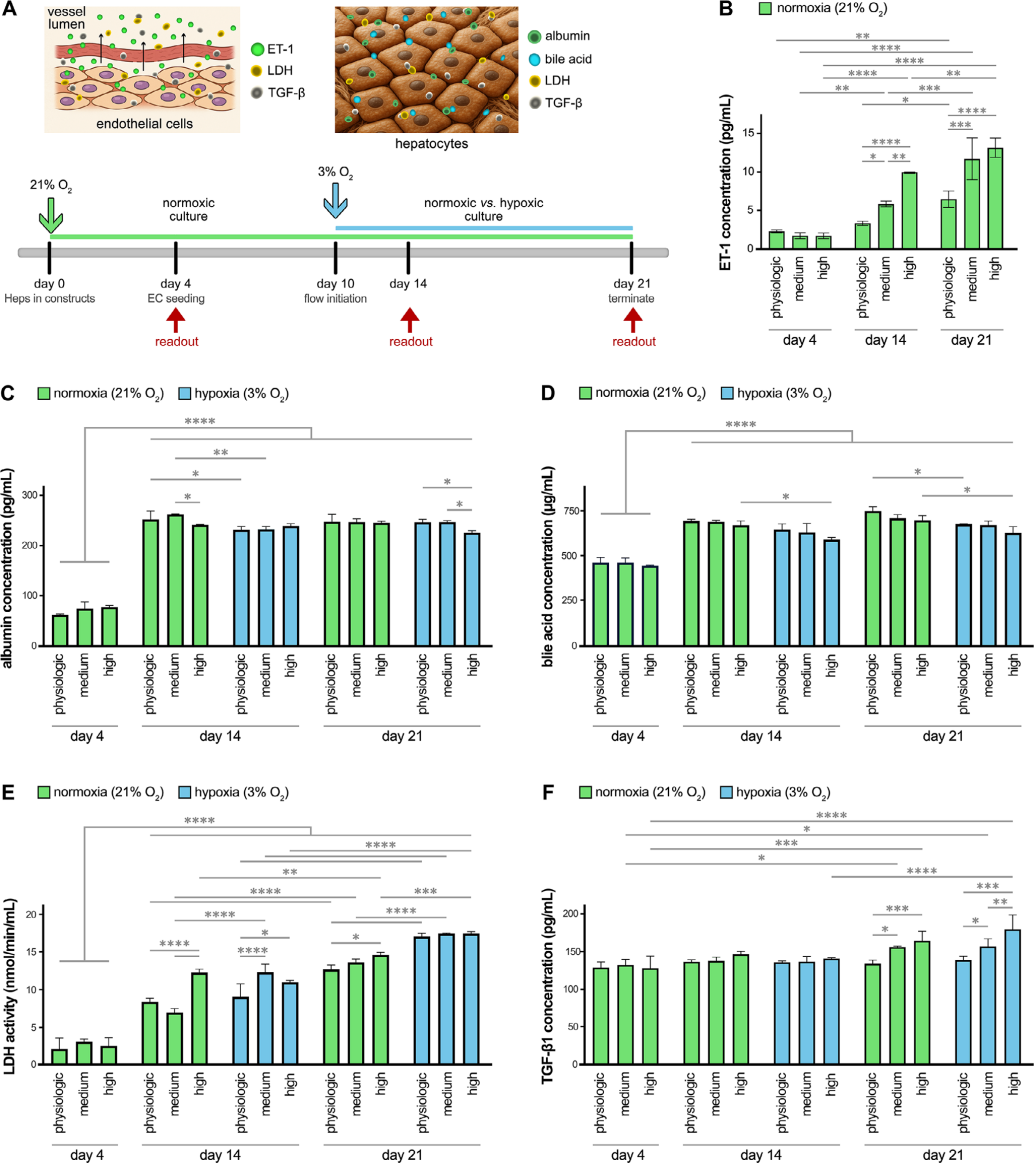
Pressure‐ and hypoxia‐dependent modulation of hepatic and endothelial cell function in 3D perfused liver sinusoid constructs. (A) Top: Schematic illustration of various markers in the culture supernatants, measured to assess endothelial cell (EC) and hepatocyte function. Bottom: The 3D culture timeline used to expose cells to normoxic (green) and hypoxic (blue) conditions. (B) Endothelin‐1 (ET‐1) secretion from endothelialized constructs under normoxic conditions showed a significant increase with rising pressure (physiologic, medium, and high pressure) and longer exposure times (day 14 vs. 21), indicating progressive EC activation and mechanosensitive stress response (*n* = 3 per group). (C–F) Albumin (C), bile acid (D), lactate dehydrogenase (LDH) (E), and TGF‐β1 (F) secretions by encapsulated hepatocytes (HepG2 cells) measured on the days 4, 14, and 21 of culture, under normoxic (21% O_2_) and hypoxic (3% O_2_) conditions (*n* = 3 per group). Data are presented as mean ± SD. Statistical significance was assessed using two‐way ANOVA followed by Tukey's multiple comparison test. *: *p* < 0.05, **: *p* < 0.01, ***: *p* < 0.001, and ****: *p* < 0.0001.

To complement our immunofluorescence findings and phenotypic analyses (Figures 6 and 7), we performed targeted metabolic profiling of culture media (supernatant) collected from constructs under differential pressure and oxygen conditions (Figure 8). These metabolic assays provided additional insight into endothelial activation, hepatic synthetic function, ECM remodeling, and cell viability in response to Fontan‐relevant mechanical and hypoxic stressors.

Consistent with the VCAM‐1 upregulation observed in endothelial layers under elevated pressure (Figure 6A,B), secretion of endothelin‐1 (ET‐1), a potent vasoconstrictor and key marker of endothelial activation, was significantly increased in normoxic groups subjected to rising perfusion pressure (Figure 8A,B). ET‐1 levels progressively increased from physiologic to high‐pressure condition (*p* < 0.0001), and also showed a time‐dependent elevation between days 14 and 21, suggesting cumulative endothelial stress over prolonged exposure. As a mechanosensitive mediator, ET‐1 is strongly associated with capillarization of LSECs, promoting defenestration and a shift toward a vasculopathic phenotype [94, 95, 96]. ET‐1 induction under high‐pressure conditions aligns with prior reports linking mechanical stress to LSEC remodeling, portal hypertension, and matrix deposition in fibrotic and congestive hepatopathies [97, 98, 99, 100]. Together with increased VCAM‐1 expression, these findings reinforce the role of elevated pressure in promoting sinusoidal capillarization and endothelial dysfunction, key pathophysiological features of the FALD.

The effects of hemodynamic and oxygen stress on hepatic function were further evaluated via albumin and bile acid secretion (Figure 8C,D). Albumin secretion progressively increased (*p* < 0.0001) from day 4 to days 14 and 21, confirming the long‐term functional viability and maturation of HepG2 cells within the 3D constructs (Figure 8C). Under 3% O_2_ conditions, albumin secretion on day 14 was modestly lower than normoxic controls. However, this difference was small in magnitude and was not consistently observed on day 21, suggesting that hypoxia alone had a limited effect on albumin production under these experimental conditions. However, within the hypoxic group on day 21, albumin secretion exhibited a modest but significant (*P*  <  0.05) decrease in the high‐pressure group compared to the physiologic and medium‐pressure conditions, suggesting that prolonged hypoxia combined with elevated pressure further attenuates hepatic synthetic output [101, 102]. These findings are consistent with the observed HIF1α upregulation (Figure 5A,B), where hypoxia triggered the nuclear accumulation of HIF1α and metabolic reprogramming in hepatocytes [86, 88, 90].

Bile acid levels in the culture media increased from day 4 to day 14 but remained unchanged in response to varying pressure conditions throughout the entire period; this is while the hypoxic groups exhibited reduced bile acid levels (Figure 8D). These findings suggest that oxygen availability is a key regulator of bile acid biosynthesis and transporter activity in this model, aligning with prior reports that highlight the sensitivity of CYP7A1 and other bile acid‐related genes to hypoxic environments [103, 104]. Collectively, this data aligns with earlier liver‐on‐chip studies and transcriptomic analyses showing that hypoxia downregulates genes involved in bile acid transport, albumin synthesis, and overall hepatic metabolism.

Beyond synthetic and metabolic markers, tissue injury and pro‐fibrotic signaling were evaluated via lactate dehydrogenase (LDH) and transforming growth factor beta (TGF‐β) secretion (Figure 8E,F). LDH release, an indicator of membrane disruption and cell injury, was significantly elevated in hypoxic groups compared to normoxic controls across all pressure levels, especially on day 21 (*p* < 0.0001) (Figure 8E). This time‐dependent increase under 3% O_2_ suggests cumulative cellular stress and damage in both hepatocyte and endothelial compartments over extended hypoxic exposure. While pressure‐related changes in LDH levels were more pronounced in normoxic conditions, showing progressive elevation from physiologic to high pressure settings, this trend was less evident under hypoxia where oxygen deprivation appeared to be the dominant stressor. These observations are in line with previous studies showing that hypoxia independently contributes to hepatocellular and endothelial injury by disrupting mitochondrial function and elevating oxidative stress [105, 106].

While albumin secretion showed only modest changes under hypoxia and pressure, increases in HIF1α and fibronectin indicate early molecular alterations and activation of stress and matrix remodeling pathways at the cellular level. This temporal dissociation may indicate that stress‐induced responses precede detectable changes in bulk synthetic function. It is also possible that the duration or intensity of stress applied in this study was sufficient to induce cellular activation and injury signals, but not severe enough to compromise overall construct‐level metabolic output. LDH release reflects membrane stress or injury from both endothelial and hepatocyte compartments and was not distinguished by cell type in this study. The increase in LDH did not correspond to a proportional decline in albumin secretion, suggesting that the observed LDH elevation reflects sublethal cellular stress rather than overt loss of hepatocyte synthetic capacity.

TGF‐β secretion, a central mediator of hepatic fibrogenesis, was not significantly elevated on day 14 under any pressure or oxygen condition (Figure 8F); however, TGF‐β levels increased notably by day 21 with rising perfusion pressure in both normoxic and hypoxic groups, with the highest expression observed under combined high‐pressure and hypoxia. This delayed but robust upregulation highlights that short‐term exposure to mechanical or hypoxic stress alone at early time points is insufficient to trigger pro‐fibrotic signaling, but the accumulation of both stressors over time synergistically promotes fibrogenic cascades. This result aligns with longitudinal studies in FALD patients, where fibrosis and associated molecular markers increase progressively over years of Fontan circulation, rather than emerging acutely post‐surgery [50]. Importantly, this fibrotic activation was most pronounced in the high‐pressure, hypoxic group on day 21, mirroring the pericentral region of the liver, where both hypoxia and venous congestion are most severe in Fontan physiology [107]. Histopathological studies have confirmed that FALD manifests primarily with pericentral fibrosis, consistent with zones of greatest mechanical and oxygen stress [108, 109]. Our results are consistent with the vulnerability of the pericentral region in FALD, where hypoxia and venous congestion are most severe. While the current model does not spatially reproduce zonation within a single construct, the combined high‐pressure and hypoxic condition reflects the microenvironment characteristic of this region. These results also support earlier findings of increased fibronectin expression under hypoxia (Figure 7H–K) and aligns with the known role of TGF‐β in driving ECM deposition, epithelial‐mesenchymal transition (EMT), and hepatic stellate cell activation [110, 111, 112].

Compared to traditional 2D cultures, liver‐on‐chip, spheroid systems, and animal models, this 3D platform is particularly valuable for mechanistic exploration of early fibrotic triggers, including hypoxia‐induced hepatocyte stress and mechanotransduction in LSECs, which are often observed in Fontan patients [14, 31]. LSECs play a central role in regulating hepatic immune responses, fenestration, and capillary dynamics [113, 114]. Under chronic congestion and hypoxia, LSECs undergo structural and phenotypic changes that exacerbate fibrosis and inflammation, which our model may recapitulate through endothelial readouts [115]. This modular platform not only supports physiologically relevant pressures and oxygen gradients but also enables the precise modeling of sinusoidal cell responses under controlled disease‐like conditions. The integration of time‐staged seeding, perfusion, and environmental cues makes this system uniquely suited for longitudinal studies of endothelial activation, hepatocyte stress, and early fibrotic signaling [33, 34, 35].

Together, our findings show that sustained exposure to both hypoxia and elevated pressure reproduces the key molecular and spatial characteristics of fibrogenesis observed in FALD. These data demonstrate that while pressure initiates endothelial and hepatocellular stress responses, hypoxia amplifies injury and triggers fibrogenic signaling, particularly when both stressors are prolonged. The synergistic responses observed under combined hypoxia and elevated pressure further confirms the mechanistic convergence of oxygen and mechanical regulation in liver disease progression, highlighting the utility of this platform for studying complex liver pathophysiologies.

## 3 Conclusion

In this study, we developed a perfusable, 3D bioengineered liver sinusoid model that recapitulates critical structural, functional, and hemodynamic features of the hepatic microenvironment. The structural fidelity of the platform was comprehensively validated using a series of geometric and mechanical measurements. SEM confirmed the preservation of lumen architecture, surface integrity, and interconnected porosity necessary for mass transport (diffusion and permeability) and cell viability. Mechanical testing demonstrated stiffness values within the physiological range of native liver tissue, supporting the model's relevance for mechanotransduction studies. Flow characterization using catheter‐based pressure wire measurements, CFD, and PIV revealed tunable and stable perfusion dynamics across physiological and pathological CVP ranges. These results confirmed the platform's ability to mimic hemodynamic environments characteristic of FALD. The construct maintained endothelial monolayer integrity and supported hepatocyte viability and function over extended perfusion culture, while responding predictably to pressure and oxygenation cues. Collectively, this model offers a robust, biomimetic system for mechanistic investigations of FALD and serves as a promising platform for therapeutic screening and precision medicine applications.

This model allowed for the decoupling of pressure and oxygen effects on both endothelial and hepatocyte compartments, revealing distinct and synergistic contributions of these variables to disease‐relevant behaviors. Endothelial markers such as VCAM‐1 and ET‐1 were upregulated under elevated pressure, reflecting early vasculopathic changes and capillarization consistent with sinusoidal remodeling in FALD. Hepatocytes showed pressure‐dependent changes in albumin and bile acid secretion, and significant hypoxia‐driven upregulation of HIF1α and fibronectin, mimicking metabolic and matrix responses observed in early fibrosis. Furthermore, hypoxic conditions led to increased LDH release, indicating hepatocellular stress and membrane damage, while TGF‐β secretion was markedly elevated under combined hypoxia and high‐pressure conditions, suggesting activation of profibrotic signaling. These findings align with recent single‐cell multi‐omics studies that suggest dynamic endothelial‐stromal‐immune crosstalk and sinusoidal microthrombi as early mediators of fibrotic remodeling in FALD [14, 32].

By integrating multicellular biofabrication, precise fluid control, and longitudinal immuno‐metabolic profiling, our platform enables dynamic modeling of hepatic responses under controlled, disease‐relevant mechanical and oxygenation conditions. This system represents a significant advancement over static or low‐fidelity models by reproducing the spatiotemporal complexities of sinusoidal physiology and pathophysiology. Together, these results demonstrate the capacity of the platform to capture early pathophysiological events in FALD progression, providing a robust system for dissecting mechanistic pathways and testing therapeutic interventions under clinically relevant stimuli.

Future directions include incorporating additional non‐parenchymal liver cell types, such as HSCs and Kupffer cells, to better recapitulate and examine the fibrotic and immune landscape of FALD. In particular, integrating HSCs will enable direct investigation of mechanosensitive stellate cell activation, ECM deposition, and progressive fibrosis, which are central drivers of FALD pathogenesis. Extending culture duration under sustained hemodynamic and hypoxic stress will further enable modeling of chronic disease progression and later‐stage fibrotic remodeling. Further miniaturization of the channel architecture toward true microscale sinusoid dimensions will enhance geometric fidelity and enable more refined modeling of sinusoidal microvascular transport phenomena. Application of 3D spatial omics and real‐time biosensing will allow deeper mechanistic insights and high‐content screening capabilities. Adaptation of the platform for patient‐derived iPSC models may enable personalized assessment of disease progression and therapeutic efficacy. Overall, this engineering‐driven, biomimetic liver sinusoid model provides a valuable platform for investigating the mechanobiology of FALD and advancing translational research in hepatic vascular disorders.

## 4 Experimental Section

### 4.1 HUVEC Culture

As described before [39, 116, 117, 118], GFP‐labeled HUVECs (ATCC) were cultured in tissue culture flasks (37°C, 5% CO_2_) using endothelial medium (LL‐0003; LIFELINE) and passaged every four to five days with 0.05% Trypsin‐EDTA (25‐300‐054; Gibco).

### 4.2 HepG2 Cell Culture

HepG2 cells were maintained in Dulbecco's modified Eagle medium (DMEM) (15017CV; Corning), supplemented with 10% fetal bovine serum (FBS) (30‐2020; ATCC), 1% penicillin‐streptomycin (P4333; Sigma Aldrich), and 1% Minimum Essential Medium (MEM) non‐essential amino acids (11140050; Gibco). The cells were cultured in tissue culture flasks coated with collagen (calf skin) (C8919; Sigma Aldrich) at 37°C in a humidified atmosphere with 5% CO_2_. Culture media was changed every 3‐4 days. When cells reached 90% confluency, they were detached using trypsin‐EDTA (Gibco).

### 4.3 Preparation of Various Bioink Solutions

Gelatin methacrylate (GelMA) was synthesized following our established protocol [19, 43, 119]. Briefly, porcine gelatin powder (G2500; Sigma Aldrich) was dissolved in phosphate‐buffered saline (PBS) at a concentration of 10% (w/v) at 50°C. Methacrylic anhydride (MA) (276685; Sigma Aldrich) was then added dropwise while stirring for 3 h at 50°C to functionalize the gelatin. To stop the reaction, additional warm PBS was added. The mixture was dialyzed against deionized water at 40°C for 1 week, with water changes 3 times daily. After purification, the GelMA solution was lyophilized for 7 days and stored at −20°C in the dark until use.

For stock solution preparation, lyophilized GelMA foam was reconstituted in sterile PBS (45000; Corning) to a concentration of 10% w/v. For the outer layer of liver constructs, containing hepatic cells, 1% w/v Irgacure 2959 (410896; Sigma Aldrich) was added. For the inner layer, containing HUVECs on the surface, 0.5% w/v lithium phenyl‐2,4,6‐trimethylbenzoylphosphinate (LAP) (900889; Millipore Sigma) was used. The reconstituted GelMA solutions were stored in the dark at 4°C and used within 1–2 weeks.

To prepare the cellular bioink solution for hepatic (outer) layer, we combined the components above to achieve final concentrations of 5% w/v GelMA, 0.5% w/v Irgacure, and 2.7 mg/mL collagen type I (rat tail) (354249; Corning). The pH was adjusted to 7.2 to 7.4. Subsequently, 30 million cells/mL HepG2 were added to the hepatic bioink.

### 4.4 Design and Biofabrication of FALD Model

The liver sinusoid model was designed to mimic the structural and functional characteristics of the human liver sinusoid, incorporating multiple cell types in a 3D configuration within a perfusable construct. The housing of the liver sinusoid was designed with computer‐aided design (CAD) software (Fusion 360, Autodesk), with 7.5 mm in width, 16.4 mm in length, and 8.5 mm in height, providing sufficient space to embed the cell‐laden hydrogel construct. The outer (hepatic) layer (minimum) thickness was 2.35 mm. The inner layer, intended as the space of Disse, for future incorporation of hepatic stellate cells (HSCs), had a thickness of 0.8 mm. This layer remained acellular in this study and had only HUVECs seeded on the surface inside. Finally, a central hollow channel at a diameter of 1.2 mm was incorporated in the center of constructs to enable perfusion, simulating the sinusoidal lumen.

The casting process was performed in two steps to form distinct layers and create a perfusable channel within the construct. In the first step, the outer layer was cast to form the hepatocyte compartment. This layer was constructed using a bioink optimized for hepatocyte function, containing 5% GelMA, 0.5% Irgacure, and 2.7 mg/mL collagen mixed with HepG2 media, along with 30 million/mL of HepG2 cells. When casting the bioink into the housing, a 3D printed (using Form 3, Formlabs) resin rod at 2.8 mm diameter was inserted in the center to keep the central channel space open. Cast hepatic tissue was crosslinked with UV light at 10 mW cm^−2^ for 2 min on both the top and bottom. The crosslinking time was optimized based on tissue stiffness requirements, ensuring that the construct would maintain structural stability while mimicking the mechanical properties of liver tissue.

Following the stabilization of the hepatic layer, the resin rod was removed, creating a hollow channel space. Next, we cast a 10% GelMA ink containing 0.5% LAP photoinitiator to create the space of Disse‐like layer. To create the perfusable lumen, another needle‐shaped resin rod (printed via Form) at a 1.2 mm diameter was placed in the center of the construct. The cast acellular GelMA layer was crosslinked with blue light at 100 mW cm^−2^ for 2 min on each side. Once the inner layer was crosslinked, the needle was gently removed, creating a central channel that served as a sinusoid‐like channel for circulating culture media.

### 4.5 Ultrastructural Analysis of Sinusoid Constructs

Pore size of the fabricated 3D scaffolds was quantified using scanning electron microscopy (SEM). Liver sinusoid constructs (*n* = 3) were washed in deionized (DI) water for 3 days. Following the washes, the samples were frozen at −80°C and lyophilized for 5 days. The freeze‐dried samples were then examined using a SEM (Axia ChemiSEM; Thermo Scientific). Pore dimensions (diameter, area, and circularity) were quantified and plotted as histograms [120, 121].

### 4.6 Micromechanical Characterization

The elastic (Young's) modulus (*E*), which reflects the stiffness of bioprinted models, was measured using the microindentation test (Mach‐1, Biomomentum) and linear contact mechanics as described before [43, 119, 122, 123, 124]. Microindentation was performed on both acellular and cellular constructs across the four outer surfaces of the 3D model and the inner surfaces of longitudinal and cross‐sectional mid‐cuts. Briefly, a microindentation tester with a 500 µm spherical probe was used to generate a load‐displacement curve. The reduced elastic modulus (*E_r_*) was derived from the unloading curves using the following formula [42].

(1) $$E_{r}=\frac{\sqrt{\pi }}{2\beta }\frac{S}{\sqrt{A\left(h_{c}\right)}}$$

where β is a constant and equals 1, and *A*(*h_c_*) is projected contact area at the contact depth of *h_c_*. It can be obtained from the following equation:

(2) $$A\left(h_{c}\right)=2\pi Rh_{c}-\pi {h_{c}}^{2}$$

where

(3) $$h_{c}=h_{max}-\varepsilon \frac{P_{max}}{S}$$

where *h_max_* and *P_max_* are the peak unloading displacement and unloading force, respectively, and ε is a constant with a value of 0.75 for a spherical indenter [125].

The Young's modulus, *E*, can be calculated using the following equation [42]:

(4) $$\frac{1}{E_{r}}=\frac{1-v^{2}}{E}+\frac{1-v_{i}^{2}}{E_{i}}$$

where *v* is the Poisson's ratio of tested material with a value of 0.5, and *v_i_* is 0.5 for the indenter tip material. *E_i_* represents the elastic modulus of the probe with a value of 2 GPa.

### 4.7 Endothelialization of Sinusoid Constructs

Four days after static culture of HepG2‐loaded 3D constructs, GFP‐labelled HUVECs were manually seeded into the luminal space of the channel in the center of each construct (10 million cells/mL). The cell suspension was slowly injected into the central lumen from the inlet, ensuring no bubble formation, until the channel was fully filled and cell suspension emerged at the outlet, confirming complete lumen occupancy; the inlet/outlet were then capped to prevent leakage during attachment. This seeding process was repeated after 3 h, with the constructs flipped 180° to promote uniform cell distribution. After completion of HUVEC seeding, the constructs were maintained in static culture for 6 more days to ensure uniform and complete cell attachment in the lumen. Next, the cellular scaffolds were either transferred to perfusion culture or kept in static culture for another 11 days. The cultures were maintained under two different oxygen conditions, with a 21% normoxic incubator used for standard conditions and a 3% hypoxic incubator used to simulate hypoxic conditions. On day 21 of in vitro culture, liver sinusoid constructs from both static and dynamic cultures, exposed to either hypoxic or normoxic environments, were harvested for further analysis.

### 4.8 Co‐Culture Media Optimization for HUVEC and HepG2 Cells

To optimize the co‐culture conditions, two separate studies were conducted for each cell line, aiming to identify a media composition that would support both cell types effectively in a shared environment. Each study included a positive control, using commercial media for each respective cell line, and a negative control, using the commercial media for the opposite cell line. Three co‐culture groups were prepared with varying ratios of HUVEC:HepG2 media: 70:30, 80:20, and 90:10. All groups were plated in triplicate (*n* = 3) to ensure statistical reliability. Optical images were captured at 10X magnification to monitor cell growth, morphology, and interactions over time. For the HUVEC study, images were taken on days 1 and 3. For the HepG2 study, images were taken on days 1 and 5. Among the three tested co‐culture ratios, the 80% HepG2 media and 20% HUVEC media showed the most balanced results for both cell types and used for all 3D co‐culture experiments.

### 4.9 Design and Assembly of Perfusion Setup

The bioreactor system included an 8‐channel peristaltic pump (IPC ISM931C, ISMATEC, IL, US) that connected to the inlet and outlet of each sinusoid construct housing. Silicone tubing (89403‐958; VWR‐Saint Gobain) connected the pump to the constructs, aligning directly with the lumen to ensure uninterrupted flow. The other end of the tubing was attached to a media reservoir, which supplied fresh culture media to the system and enabled consistent circulation through the structures. Flow rates of 0.12 mL/min, for healthy (physiologic) condition, 0.3 mL/min for moderate FALD condition, and 0.4 mL/min for severe FALD condition were used. To increase flow resistance in the disease conditions, an adjustable mini flow control tube clamp (710667099612; Quickun) was applied on the tubing 5 cm after the outlet. This clamp enabled precise control of flow resistance to mimic the increased vascular pressure conditions observed in FALD.

### 4.10 In‐Situ Pressure Measurements

To accurately measure the lumen pressure within the liver sinusoid constructs, pressure measurements were conducted in a catheterization (cath) lab using a Volcano pressure wire (10185P: Verrata PLUS Pressure Guide Wire—Straight Tip, 0.014″ x 185 cm). This setup allowed for precise assessment of the relationship between perfusion flow rates and intraluminal pressure, simulating physiological and pathological conditions within the constructs. For this purpose, the pressure wire was inserted into the lumen of each construct, aligned with the flow path. Measurements were taken both with and without clamping the tubing downstream from the construct to simulate outflow resistance, which is characteristic of increased vascular pressure in FALD disease states. The flow rate in perfusion the bioreactor was incrementally adjusted from 0.12 mL/min up to 4.0 mL/min. Pressure readings were recorded at each flow rate. Data was analyzed to correlate flow rate with pressure increase, resulting in a detailed flow‐pressure curve.

### 4.11 In Vitro Setup and Flow Analysis using PIV

We used an Ultimaker 2+ 3D printer (Ultimaker, Geldermalsen, Netherlands) and sacrificial polyvinyl alcohol (PVA) ink to create a channel analogue, which was scaled up to 4X to enhance flow visualization and ensure compatibility with the in vitro setup. The 1X bioprinted models were too small and nontransparent, limiting the effectiveness of camera configuration and vector analysis. Therefore, a 4X scaled‐up design of the sinusoid model was fabricated by casting transparent polydimethylsiloxane (PDMS) (Dow Corning, Sylgard‐184) around the printed PVA structure. The PVA cylinder was placed inside a plastic mold (matching the 4X geometry), with inlets and outlets connected to tubing. The PDMS was cured and stored for 10 h at 4°C. Subsequently, water was perfused through the construct to gradually dissolve the sacrificial PVA, resulting in a hollow channel within the transparent PDMS structure.

For PIV imaging, flow rates were varied to simulate different physiological and disease conditions. The *Hagen‐Poiseuille* equation was used to determine compatible flow rates for the 4X scaled model, using the following formula:

(5) $$Q=\frac{\pi \Delta Pr^{4}}{8\eta }$$

For baseline (healthy) flow conditions, a flow rate of 7.68 mL/min was employed, using a syringe pump (PHD 2000, Harvard Apparatus, Holliston, MA). For moderate and severe FALD conditions, flow rates increased to 19.2 mL/min and 25.6 mL/min, respectively. Additionally, a flow control clamp was applied downstream of the construct to introduce resistance. A beaker was used as the fluid reservoir and an ultrasonic flow meter (ME 4PXL, Transonic Systems) downstream of it was used to monitor the flow rate continuously. Pressure measurements were performed upstream and downstream of the model using a Millar catheter (ADInstruments Inc). A peristaltic pump (VWR Variable‐Speed, USA) was used to drive the flow through the inlet. The outlet port returned the fluid to the beaker, closing the flow loop. The model was placed horizontally in a tank filled with PBS to minimize optical distortions. With the kinematic viscosity of the working solution (0.9‐1.1 cSt at 20°C) close to that of water (1 cSt at 20°C) and the model four times larger, the Reynolds numbers were closely matched in both experiments. An Nd:YLF laser (DM20‐527‐DH, Photonics Lasers) beam was delivered by an articulated arm (LaVision 1108453) and converted to a thin sheet (less than 1 mm) by an adjustable sheet optics (LaVision 1108405). The laser sheet cut through the centerline of the flow channels in the PDMS model.

The circulating fluid was loaded with 2 µm polystyrene particles coated with Rhodamine 6G (LaVision 1001851). A CMOS high‐speed camera (Phantom VEO‐E 340L, 2560 × 1600 px, AMETEK) with a Nikon NIKKOR 105 mm Macro lens was used to record the particle fields through a 45° mounted front surface mirror [126]. A long‐pass filter (>540 nm, LaVision 1108573) mounted in front of the lens was used to block the scattered laser light by the model. The field‐of‐view was 76 × 47 mm A total of 2000 frames of particle images were recorded at 150 frames per second and the corresponding Δ*t*. The Δ*t* between images was 6.67 ms [126]. The maximum particle displacement between frames was 6 pixels. The particle images were processed initially by background removal, followed by a multipass cross‐correlation algorithm (LaVision Davis 10). With a final interrogation window size of 16 × 16 pixels and a 50% overlap, the resulting vector spacing was 0.24 mm, corresponding to 33 vectors across the diameter of the channel. A universal outlier detection algorithm was applied to remove the spurious vectors [127]. The uncertainty of the measured velocity was around 0.2 mm/s.

### 4.12 Computational Fluid Dynamics (CFD) Modeling of Flow Hemodynamics

For CFD simulations, the CAD model of vascular construct was created using Autodesk Fusion 360. Time dependent CFD simulations under a laminar flow assumption (𝜌 = 1060 kg/m^3^) were carried out using FLUENT solver (ANSYS Inc., Lebanon, NH, USA). A volumetric flow waveform mapped to a parabolic profile and a low constant pressure were used for inlet and outlet boundaries, respectively. For medium and high‐pressure groups, a high resistance applied after the outlet. A rigid no‐slip boundary condition was used at the entire wall sections of the model. CFD simulations were conducted at two scales: 1) the scale used for laser PIV analyses, referred to as 4X, with inlet and outlet being 4.8 mm in diameter; and 2) the scale used for bioprinting (1X). The 4X simulations were used for assessing CFD predictions against PIV measurements (performed at the same scales), while 1X simulations, performed at a similar Reynold number, enabling us to estimate flow patterns and relevant hemodynamic factors that existed in the bioprinting samples. The pulsatile volumetric flow waveform was scaled to provide average inlet velocity ranging between 0 to 5 mm/s (for 4X), which represented a common flow pattern in the liver sinusoids. For all simulations, fluid was considered Newtonian with a dynamic viscosity of 1.3 mPa.s, consistent with the fluid properties used in PIV. Anisotropic discretization with tetrahedral elements was used for each model with a characteristic element length of 0.7 mm at the center and 0.45 mm at the first four layers adjacent to the wall (for 4X). Each simulation was conducted for multiple cycles (using 1000‐time steps per cycle), mesh convergence was verified, and the last cycle was used for analysis. In addition to calculating the velocity field, values of pressure within the lumen were analyzed to predict the pressure distribution along the channel. The pressure drop between the inlet and outlet boundaries was used as a metric for assessing hemodynamic resistance within the conduit. This pressure‐based approach provided insights into the overall flow behavior and the hydraulic performance of the vascular constructs, which are critical for maintaining physiological and disease conditions in the bioprinted models.

### 4.13 Metabolomic Assays

For the analysis of bile acid, albumin and endothelin‐1, supernatant media samples were collected from each culture condition at three time points: days 4, 14, and 21. After collection, all samples were immediately stored at −80°C to preserve stability and prevent degradation until further analysis. Each construct was maintained in 30 mL of media throughout the experiment (changed every 4 days). Prior to each assay, samples were thawed and prepared according to the protocol specified for each respective assay.

For bile acid quantification, a colorimetric assay was performed. Following the protocol provided in the bile acid kit (ab239702; Abcam), each thawed sample was mixed with a chromogenic substrate that specifically reacts with bile acids to produce a color change. This colorimetric reaction was measured at an absorbance of 405 nm using a microplate reader. Bile acid concentrations in each sample were calculated by comparing the absorbance readings to a standard curve generated from known concentrations of bile acid standards. Assays were performed in duplicate to ensure accuracy.

Albumin and endothelin‐1 levels were quantified using ELISA kits (ab108788 and ab133030, Abcam). Samples were added to the wells pre‐coated with albumin or endothelin‐1 specific antibodies. After an incubation period, unbound components were washed away, and a biotinylated detection antibody (specific to albumin or endothelin‐1) was added. Following another wash, streptavidin conjugated to horseradish peroxidase (HRP) was introduced, and a chromogenic substrate was applied to develop a color change. The absorbance was read immediately at 450 nm, with correction between 570 and 590 nm. Albumin and endothelin‐1 concentrations were determined by interpolation from a standard curve.

TGF‐β1 levels were measured using the human TGF‐β1 ELISA kit (ab100647; Abcam). Prior to the assay, latent TGF‐β1 in the samples was activated using acidification with 1 N HCl followed by neutralization with 1.2 N NaOH/0.5 M HEPES. Activated samples and standards were added to the wells coated with anti‐human TGF‐β1 antibodies and incubated. Following washes, biotinylated detection antibodies and HRP‐conjugated streptavidin were applied, followed by TMB substrate and stop solution. Absorbance was measured at 450 nm and TGF‐β1 concentrations were determined using a standard curve.

LDH activity in the culture media was assessed using a colorimetric LDH assay kit (ab102526; Abcam). Thawed samples were diluted and mixed with a reaction mix containing assay buffer and LDH substrate. Absorbance was measured at 450 nm in kinetic mode every 3 min at 37°C for 30 min. The LDH activity was calculated based on the rate of change in absorbance over time, using a NADH standard curve.

### 4.14 Immunohistochemical (IHC) Analysis

Samples were fixed using 4% paraformaldehyde (PFA) for 1–2 h at 4°C, ensuring immediate fixation for those maintained under hypoxic conditions. After fixation, samples were washed three times with PBS and stored in PBS at 4°C until sectioning. Samples were embedded in 4% agarose (16520050; ThermoFisher) and sectioned into approximately 300 µm‐thick sections using a vibratome (VT 1200, Leica BioSystems). Sections were stored in PBS until ready for staining.

For staining, sections were first permeabilized with 0.5% Triton X‐100 (IB07100; IBI Scientific) for 20 min at room temperature to allow antibody penetration. Following permeabilization, samples were washed with PBS and blocked for 1 h at room temperature in blocking buffer containing 1% bovine serum albumin (BSA) (PI37525; VWR International) to minimize nonspecific binding. After blocking, primary antibodies, each diluted to 1:200 in PBS, were added to the sections and incubated overnight on a rocker at 4°C. The primary antibodies used for HUVECs included anti‐GFP (GFP‐1020; Aves Lab), anti‐CD105 (ab2529; Abcam), and anti‐VCAM‐1 (ab134047; Abcam). For HepG2 cells we used anti‐HNF‐4α (ab41898; Abcam) and anti‐Fibronectin (ab2413; Abcam). The anti‐HIF‐1α (ab51608; Abcam) was used as a hypoxia marker. Following primary antibody incubation, the sections were washed three times for 5 min each with PBS at room temperature. Secondary antibodies including Alexa Fluor 488 (ab150169; Abcam), Alexa Fluor 555 (A‐21428; ThermoFisher), Alexa Fluor 647 (A‐31571; ThermoFisher), and DAPI (D1306; Fisher Scientific), diluted to 1:500 in PBS, were added to the sections, and samples were incubated for 2 h at room temperature, protected from light. Next, sections were washed three more times for 5 min each with PBS at room temperature.

The stained samples were mounted onto microscope slides and allowed to air dry for 5–10 min. Mounting media (H‐1700‐10; Vector Laboratories) was added to the slides, which were then covered with coverslips. The slides were allowed to dry for 24–48 h and stored at 4°C until ready for imaging.

### 4.15 Statistical Analysis

All experimental data were processed and presented using means ± standard error of means (SEM). The number of biological replicates for each experiment is indicated in the corresponding figure legend. No data transformation or normalization was applied prior to statistical analysis, and datasets were screened for outliers using the ROUT method (Q = 1%). Identified outliers were excluded from further analysis. Fluorescence intensities from immunostaining images were quantified using Leica LAS X imaging software and normalized to the DAPI signal where applicable. Statistical significance was determined by one‐way or two‐way analysis of variance (ANOVA) with Tukey's multiple comparison post‐hoc test. Statistical analyses were performed using GraphPad Prism GraphPad 10.2.3. All tests were two‐sided, and a significance level of *α* = 0.05 was used for all analyses. The statistical tests used for each experiment are indicated in figure legends. *: *p* < 0.05, **: *p* < 0.01, ***: *p* < 0.001, and ****: *p* < 0.0001.

## Author Contributions

S.R. and V.S. conceived the project and the design of the experiments. S.R. and M.S.A. performed the in vitro 3D model experiments and the analyses. M.S.A., B.H., and L.J. helped with the perfusion assays and analysis of 3D FALD constructs. T.M. and R.A. performed the CFD analysis and validation. M.B., S.K., and L.P.D. performed PIV experiments and analyses. S.R. and M.S. helped with the PIV experiments. S.R., Y.S., S.N., and A.V.B. performed micro/macro fidelity, micromechanical, and pore size analyses. S.R. led the immunohistochemistry analyses and L.J., B.H., and S.N. helped with the processes. H.D.B. led the works related to pressure measurement in the cath lab. H.D.B., R.R., L.P.D., R.A., and V.S. provided critical expertise and resources. S.R. and V.S. prepared the original and revised versions of the manuscript. All authors reviewed and approved the manuscript.

## Conflicts of Interest

The authors declare no conflicts of interest.

## Supporting information




**Supporting File**: advs74851‐sup‐0001‐SuppMat.docx.

## Data Availability

The data that support the findings of this study are available from the corresponding author upon reasonable request.

## References

[1] J. R. Dillman , A. T. Trout , T. Alsaied , A. Gupta , and A. M. Lubert , “Imaging of Fontan‐Associated Liver Disease,” Pediatric Radiology 50, no. 11 (2020): 1528–1541, 10.1007/s00247-020-04776-0. 32809067

[2] A. Prakash , A. J. Powell , and T. Geva , “Multimodality Noninvasive Imaging for Assessment of Congenital Heart Disease,” Circulation: Cardiovascular Imaging 3, no. 1 (2010): 112–125, 10.1161/CIRCIMAGING.109.875021. 20086225

[3] M. E. Oster , J. H. Knight , D. Suthar , O. Amin , and L. K. Kochilas , “Long‐Term Outcomes in Single‐Ventricle Congenital Heart Disease,” Circulation 138, no. 23 (2018): 2718–2720. 30571273 10.1161/CIRCULATIONAHA.118.036821 PMC6309811

[4] L. S. Kverneland , P. Kramer , and S. Ovroutski , “Five Decades of the Fontan Operation: a Systematic Review of International Reports on Outcomes after Univentricular Palliation,” Congenital Heart Disease 13, no. 2 (2018): 181–193, 10.1111/chd.12570. 29372588

[5] T. B. Fredenburg , T. R. Johnson , and M. D. Cohen , “The Fontan Procedure: Anatomy, Complications, and Manifestations of Failure,” Radiographics 31, no. 2 (2011): 453–463, 10.1148/rg.312105027. 21415190

[6] J. Emamaullee , A. N. Zaidi , T. Schiano , et al., “Fontan‐Associated Liver Disease: Screening, Management, and Transplant Considerations,” Circulation 142, no. 6 (2020): 591–604. 32776846 10.1161/CIRCULATIONAHA.120.045597 PMC7422927

[7] C. de Lange , T. Möller , and H. Hebelka , “Fontan‐associated Liver Disease: Diagnosis, Surveillance, and Management,” Frontiers in Pediatrics 11 (2023), 10.3389/fped.2023.1100514. PMC10020358 36937979

[8] N. Perrin , A. Dore , A. van de Bruaene , et al., “The Fontan Circulation: from Ideal to Failing Hemodynamics and Drug Therapies for Optimization,” Canadian Journal of Cardiology 38, no. 7 (2022): 1059–1071. 35469974 10.1016/j.cjca.2022.04.014

[9] A. C. Egbe , H. M. Connolly , W. R. Miranda , et al., “Hemodynamics of Fontan Failure: The Role of Pulmonary Vascular Disease,” Circulation: Heart Failure 10, no. 12 (2017): 004515. 10.1161/CIRCHEARTFAILURE.117.004515 PMC5739063 29246897

[10] G. A. Mazza , E. Gribaudo , and G. Agnoletti , “The Pathophysiology and Complications of Fontan Circulation,” Acta Biomed 92, no. 5 (2021): 2021260. 10.23750/abm.v92i5.10893 PMC8689331 34738582

[11] M. B. Hilscher and P. S. Kamath , “Fontan‐Associated Liver Disease,” Clinical Liver Disease 22, no. 4 (2023): 130–133, 10.1097/CLD.0000000000000061. 37908864 PMC10615527

[12] A. Cieplucha , W. Budts , M. Gewillig , and A. V. D. Bruaene , “Fontan‐associated Liver Disease in Adults: What a Cardiologist Needs to Know. A Comprehensive Review for Clinical Practitioners,” US Cardiology Review 16 (2022): 25, 10.15420/usc.2022.02. PMC11588172 39600838

[13] M. B. Hilscher , M. L. Wells , S. K. Venkatesh , F. Cetta , and P. S. Kamath , “Fontan‐Associated Liver Disease,” Hepatology 75, no. 5 (2022): 1300–1321, 10.1002/hep.32406. 35179797

[14] M. B. Hilscher , T. Sehrawat , J. P. Arab , et al., “Mechanical Stretch Increases Expression of CXCL1 in Liver Sinusoidal Endothelial Cells to Recruit Neutrophils, Generate Sinusoidal Microthombi, and Promote Portal Hypertension,” Gastroenterology 157, no. 1 (2019): 193–209.e9, 10.1053/j.gastro.2019.03.013. 30872106 PMC6581607

[15] F. W. DiPaola , K. R. Schumacher , C. S. Goldberg , J. Friedland‐Little , A. Parameswaran , and J. R. Dillman , “Effect of Fontan Operation on Liver Stiffness in Children with Single Ventricle Physiology,” European Radiology 27, no. 6 (2017): 2434–2442, 10.1007/s00330-016-4614-x. 27752831

[16] B. Chen , R. A. Schreiber , D. G. Human , J. E. Potts , and O. R. Guttman , “Assessment of Liver Stiffness in Pediatric Fontan Patients Using Transient Elastography,” Canadian Journal of Gastroenterology and Hepatology 2016 (2016): 7125193. 27656638 10.1155/2016/7125193 PMC5021462

[17] M. A. Padalino , L. Chemello , L. Cavalletto , A. Angelini , and M. Fedrigo , “Prognostic Value of Liver and Spleen Stiffness in Patients with Fontan Associated Liver Disease (FALD): a Case Series with Histopathologic Comparison,” Journal of Cardiovascular Development and Disease 8, no. 3 (2021): 30. 33809668 10.3390/jcdd8030030 PMC8002245

[18] T. Nagasawa , H. Kuroda , T. Abe , H. Saiki , and Y. Takikawa , “Shear Wave Dispersion to Assess Liver Disease Progression in Fontan‐associated Liver Disease,” PLoS ONE 17, no. 7 (2022): 0271223, 10.1371/journal.pone.0271223. PMC9269959 35802664

[19] A. D. Cetnar , M. L. Tomov , L. Ning , et al., “Patient‐Specific 3D Bioprinted Models of Developing Human Heart,” Advanced Healthcare Materials 10 (2020): 2170071. 10.1002/adhm.202001169 PMC8175477 33274834

[20] D. H. Bang , Y. Son , Y. H. Lee , and K. H. Yoon , “Doppler Ultrasonography Measurement of Hepatic Hemodynamics during Valsalva Maneuver: Healthy Volunteer Study,” Ultrasonography 34, no. 1 (2015): 32–38. 25327526 10.14366/usg.14029 PMC4282232

[21] J. Fan , C. J. Chen , Y. C. Wang , W. Quan , J. W. Wang , and W. G. Zhang , “Hemodynamic Changes in Hepatic Sinusoids of Hepatic Steatosis Mice,” World Journal of Gastroenterology 25, no. 11 (2019): 1355–1365, 10.3748/wjg.v25.i11.1355. 30918428 PMC6429340

[22] H. Malhi , R. S. Brown Jr. , J. K. Lim , et al., “Precipitous Changes in Nomenclature and Definitions—NAFLD Becomes SLD: Implications for and Expectations of AASLD Journals,” Hepatology Communications 7, no. 11 (2023): 0318, 10.1097/HC9.0000000000000318. PMC10635597 37941420

[23] S. Wang and S. L. Friedman , “Found in Translation—Fibrosis in Metabolic Dysfunction–Associated Steatohepatitis (MASH),” Science Translational Medicine 15, no. 716 (2023): adi0759, 10.1126/scitranslmed.adi0759. PMC10671253 37792957

[24] Y. Zongtao , W. Huishan , W. Zengwei , et al., “Experimental Study of Nonpulsatile Flow Perfusion and Structural Remodeling of Pulmonary Microcirculation Vessels,” The Thoracic and Cardiovascular Surgeon 58, no. 8 (2010): 468–472, 10.1055/s-0030-1250124. 21110269

[25] R. Henaine , M. Vergnat , E. A. Bacha , et al., “Effects of Lack of Pulsatility on Pulmonary Endothelial Function in the Fontan Circulation,” The Journal of Thoracic and Cardiovascular Surgery 146, no. 3 (2013): 522–529, 10.1016/j.jtcvs.2012.11.031. 23219498

[26] J. Wu , W. Zhou , L. Wu , Y. Qian , Y. Lu , and F. Li , “Ionic Mechanisms Underlying Atrial Electrical Remodeling after a Fontan‐Style Operation in a Canine Model,” Heart and Vessels 35, no. 5 (2020): 731–741, 10.1007/s00380-019-01544-5. 31912231 PMC7136189

[27] Z. Jalal , E. Langouet , N. Dib , et al., “Role and Applications of Experimental Animal Models of Fontan Circulation,” Journal of Clinical Medicine 13, no. 9 (2024): 2601, 10.3390/jcm13092601. 38731130 PMC11084605

[28] H. Higashiyama , M. Yamaguchi , K. Kumada , H. Sasaki , T. Yamaguchi , and K. Ozawa , “Functional Deterioration of the Liver by Elevated Inferior Vena Cava Pressure: a Proposed Upper Safety Limit of Pressure for Maintaining Liver Viability in Dogs,” Intensive Care Medicine 20, no. 2 (1994): 124–129, 10.1007/BF01707667. 8201092

[29] J. Van Puyvelde , F. Rega , T. Minami , et al., “Creation of the Fontan Circulation in Sheep: a Survival Model,” Interactive CardioVascular and Thoracic Surgery 29, no. 1 (2019): 15–21, 10.1093/icvts/ivz022. 30789218

[30] L. Boni , T. Sasaki , W. T. Ferrier , et al., “Challenges in Longer‐Term Mechanical Support of Fontan Circulation in Sheep,” ASAIO Journal 58, no. 1 (2012): 60–64, 10.1097/MAT.0b013e31823c0aa4. 22210652

[31] J. M. Kelly , Z. Hu , F. Takaesu , et al., “Investigation of a Chronic Single‐Stage Sheep Fontan Model,” JTCVS Open 21 (2024): 268–278. 39534321 10.1016/j.xjon.2024.06.018 PMC11551305

[32] P. Hu , J. Rychik , J. Zhao , et al., “Single‐Cell Multiomics Guided Mechanistic Understanding of Fontan‐Associated Liver Disease,” Science Translational Medicine 16, no. 744 (2024): adk6213, 10.1126/scitranslmed.adk6213. PMC11103255 38657025

[33] C. Lin and S. R. Khetani , “Advances in Engineered Liver Models for Investigating Drug‐Induced Liver Injury,” BioMed Research International 2016 (2016): 1829148. 27725933 10.1155/2016/1829148 PMC5048025

[34] C. Xiang , Y. Du , G. Meng , et al., “Long‐Term Functional Maintenance of Primary Human Hepatocytes In Vitro,” Science 364, no. 6438 (2019): 399–402, 10.1126/science.aau7307. 31023926

[35] P. Ros‐Tarraga , E. Villanueva‐Badenas , E. Sanchez‐Gonzalez , G. Gallego‐Ferrer , M. T. Donato , and L. Tolosa , “Challenges of In Vitro Modelling of Liver Fibrosis,” Frontiers in Cell and Developmental Biology 13 (2025): 1567916, 10.3389/fcell.2025.1567916. 40371390 PMC12075197

[36] J. Gracia‐Sancho , G. Marrone , and A. Fernández‐Iglesias , “Hepatic Microcirculation and Mechanisms of Portal Hypertension,” Nature Reviews Gastroenterology & Hepatology 16, no. 4 (2019): 221–234, 10.1038/s41575-018-0097-3. 30568278

[37] J. H. Ha , J. H. Lim , J. W. Kim , et al., “Conductive GelMA‐Collagen‐AgNW Blended Hydrogel for Smart Actuator,” Polymers 13, no. 8 (2021): 1217. 33918789 10.3390/polym13081217 PMC8068890

[38] R. Leu Alexa , H. Iovu , J. Ghitman , et al., “3D‐Printed Gelatin Methacryloyl‐Based Scaffolds with Potential Application in Tissue Engineering,” Polymers 13, no. 5 (2021): 727. 33673486 10.3390/polym13050727 PMC7956788

[39] M. S. Amoli , S. Rezapourdamanab , L. Jin , et al., “Protocol for 3D Bioprinting of a 3D in Vitro Model of Neuroblastoma,” STAR Protocols 6, no. 2 (2025): 103725, 10.1016/j.xpro.2025.103725. 40202843 PMC12008580

[40] A. G. Cinar , I. Munir , and G. Yesiloz , “Investigating Physical Properties of Hybrid Hyaluronic Acid and Collagen Compositions of GelMA Microgels Toward Tissue Engineering and Organ‐on‐Chip Applications,” ACS Applied Polymer Materials 5, no. 10 (2023): 8121–8132, 10.1021/acsapm.3c01309.

[41] Q. Hu , M. A. Torres , H. Pan , S. L. Williams , and M. Ecker , “Precision Engineering of Chondrocyte Microenvironments: Investigating the Optimal Reaction Conditions for Type B Gelatin Methacrylate Hydrogel Matrix for TC28a2 Cells,” Journal of Functional Biomaterials 15, no. 3 (2024): 77, 10.3390/jfb15030077. 38535270 PMC10971347

[42] W. C. Oliver and G. M. Pharr , “Measurement of Hardness and Elastic Modulus by Instrumented Indentation: Advances in Understanding and Refinements to Methodology,” Journal of Materials Research 19, no. 1 (2004): 3–20, 10.1557/jmr.2004.19.1.3.

[43] L. Ning , R. Mehta , C. Cao , et al., “Embedded 3D Bioprinting of Gelatin Methacryloyl‐Based Constructs with Highly Tunable Structural Fidelity,” ACS Applied Materials & Interfaces 12, no. 40 (2020): 44563–44577, 10.1021/acsami.0c15078. 32966746

[44] K. T. Suk , “Hepatic Venous Pressure Gradient: Clinical Use in Chronic Liver Disease,” Clinical and Molecular Hepatology 20, no. 1 (2014): 6–14, 10.3350/cmh.2014.20.1.6. 24757653 PMC3992331

[45] A. Schlegel , H. Mergental , C. Fondevila , R. J. Porte , P. J. Friend , and P. Dutkowski , “Machine Perfusion of the Liver and Bioengineering,” Journal of Hepatology 78, no. 6 (2023): 1181–1198, 10.1016/j.jhep.2023.02.009. 37208105

[46] A. C. Egbe , W. R. Miranda , G. R. Veldtman , R. P. Graham , and P. S. Kamath , “Hepatic Venous Pressure Gradient in Fontan Physiology Has Limited Diagnostic and Prognostic Significance,” CJC Open 2, no. 5 (2020): 360–364, 10.1016/j.cjco.2020.04.011. 32995721 PMC7499375

[47] J. Willemse , M. M. A. Verstegen , A. Vermeulen , et al., “Fast, Robust and Effective Decellularization of Whole human Livers Using Mild Detergents and Pressure Controlled Perfusion,” Materials Science and Engineering: C 108 (2020): 110200, 10.1016/j.msec.2019.110200. 31923991

[48] D. Emerson , Y. Rabin , and L. B. Kara , “A Simplified Computational Liver Perfusion Model, with Applications to Organ Preservation,” Scientific Reports 15, no. 1 (2025): 2178, 10.1038/s41598-025-85170-4. 39820266 PMC11739513

[49] G. Xu , F. Li , and Y. Mao , “Portal Pressure Monitoring—State‐of‐the‐art and Future Perspective,” Annals of Translational Medicine 7, no. 20 (2019): 583, 10.21037/atm.2019.09.22. 31807564 PMC6861775

[50] M. Gewillig and S. C. Brown , “The Fontan Circulation after 45 Years: Update in Physiology,” Heart 102, no. 14 (2016): 1081–1086, 10.1136/heartjnl-2015-307467. 27220691 PMC4941188

[51] H. Park , E. Yeom , S.‐J. Seo , J.‐H. Lim , and S.‐J. Lee , “Measurement of Real Pulsatile Blood Flow Using X‐ray PIV Technique with CO_2_ Microbubbles,” Scientific Reports 5, no. 1 (2015): 8840, 10.1038/srep08840. 25744850 PMC4351547

[52] J. Poisson , S. Lemoinne , C. Boulanger , et al., “Liver Sinusoidal Endothelial Cells: Physiology and Role in Liver Diseases,” Journal of Hepatology 66, no. 1 (2017): 212–227, 10.1016/j.jhep.2016.07.009. 27423426

[53] A. M. Malek , S. L. Alper , and S. Izumo , “Hemodynamic Shear Stress and Its Role in Atherosclerosis,” Jama 282, no. 21 (1999): 2035–2042, 10.1001/jama.282.21.2035. 10591386

[54] K. Nishikata , K. Doi , N. Kaneoya , M. Nakamura , and N. Futai , “Vitro Model of Vascular Remodeling under Microfluidic Perfusion,” Micromachines 16, no. 1 (2025): 14. 10.3390/mi16010014 PMC11767722 39858670

[55] J. W. Van der Kindere , A. Laskari , B. Ganapathisubramani , and R. de Kat , “Pressure from 2D Snapshot PIV,” Experiments in Fluids 60, no. 2 (2019): 32, 10.1007/s00348-019-2678-5. 30880869 PMC6394750

[56] P. Vennemann , R. Lindken , and J. Westerweel , “In Vivo Whole‐field Blood Velocity Measurement Techniques,” Experiments in Fluids 42, no. 4 (2007): 495–511.

[57] U. N. M. Fareez , S. A. A. Naqvi , M. Mahmud , and M. Temirel , “Computational Fluid Dynamics (CFD) Analysis of Bioprinting,” Advanced Healthcare Materials 13, no. 20 (2024): 2400643. 10.1002/adhm.202400643 38648623

[58] L. van de Velde , M. van Helvert , S. Engelhard , et al., “Validation of Ultrasound Velocimetry and Computational Fluid Dynamics for Flow Assessment in Femoral Artery Stenotic Disease,” Journal of Medical Imaging 11, no. 3 (2024): 037001. 38765874 10.1117/1.JMI.11.3.037001 PMC11097197

[59] T. Wang , S. Lü , Y. Hao , Z. Su , M. Long , and Y. Cui , “Influence of Microflow on Hepatic Sinusoid Blood Flow and Red Blood Cell Deformation,” Biophysical Journal 120, no. 21 (2021): 4859–4873, 10.1016/j.bpj.2021.09.020. 34536388 PMC8595567

[60] Y. Li , C. Puelz , M. S. Olufsen , and A. Taylor‐LaPole , “A One Dimensional (1D) Computational Fluid Dynamics Study of Fontan‐Associated Liver Disease (FALD),” International Journal for Numerical Methods in Biomedical Engineering 42 (2025): 70128. 10.1002/cnm.70128 PMC12791198 41521410

[61] E. Felli , S. Selicean , S. Guixé‐Muntet , et al., “Mechanobiology of Portal Hypertension,” JHEP Reports 5, no. 11 (2023): 100869, 10.1016/j.jhepr.2023.100869. 37841641 PMC10568428

[62] M. Casanova‐Acebes , E. Dalla , A. M. Leader , et al., “Tissue‐resident Macrophages Provide a Pro‐tumorigenic Niche to Early NSCLC Cells,” Nature 595, no. 7868 (2021): 578–584, 10.1038/s41586-021-03651-8. 34135508 PMC8923521

[63] S. R. Khetani and S. N. Bhatia , “Engineering Tissues for in Vitro Applications,” Current Opinion in Biotechnology 17, no. 5 (2006): 524–531, 10.1016/j.copbio.2006.08.009. 16978857

[64] L. D. DeLeve , “Liver Sinusoidal Endothelial Cells in Hepatic Fibrosis,” Hepatology 61, no. 5 (2015): 1740–1746, 10.1002/hep.27376. 25131509 PMC4333127

[65] B. Xu , U. Broome , M. Uzunel , et al., “Capillarization of Hepatic Sinusoid by Liver Endothelial Cell‐Reactive Autoantibodies in Patients with Cirrhosis and Chronic Hepatitis,” The American Journal of Pathology 163, no. 4 (2003): 1275–1289, 10.1016/S0002-9440(10)63487-6. 14507637 PMC1868294

[66] P. F. Lalor , W. K. Lai , S. M. Curbishley , S. Shetty , and D. H. Adams , “Human Hepatic Sinusoidal Endothelial Cells Can be Distinguished by Expression of Phenotypic Markers Related to Their Specialised Functions in Vivo,” World Journal of Gastroenterology 12, no. 34 (2006): 5429–5439, 10.3748/wjg.v12.i34.5429. 17006978 PMC4088223

[67] P. Lu , D. Ruan , M. Huang , et al., “Harnessing the Potential of Hydrogels for Advanced Therapeutic Applications: Current Achievements and Future Directions,” Signal Transduction and Targeted Therapy 9, no. 1 (2024): 166, 10.1038/s41392-024-01852-x. 38945949 PMC11214942

[68] N. Ricard , S. Bailly , C. Guignabert , and M. Simons , “The Quiescent Endothelium: Signalling Pathways Regulating Organ‐specific Endothelial Normalcy,” Nature Reviews Cardiology 18, no. 8 (2021): 565–580, 10.1038/s41569-021-00517-4. 33627876 PMC7903932

[69] J. M. Cook‐Mills , M. E. Marchese , and H. Abdala‐Valencia , “Vascular Cell Adhesion Molecule‐1 Expression and Signaling during Disease: Regulation by Reactive Oxygen Species and Antioxidants,” Antioxidants & Redox Signaling 15, no. 6 (2011): 1607–1638, 10.1089/ars.2010.3522. 21050132 PMC3151426

[70] J. J. Chiu , P. L. Lee , C. N. Chen , et al., “Shear Stress Increases ICAM‐1 and Decreases VCAM‐1 and E‐Selectin Expressions Induced by Tumor Necrosis Factor‐α in Endothelial Cells,” Arteriosclerosis, Thrombosis, and Vascular Biology 24, no. 1 (2004): 73–79, 10.1161/01.ATV.0000106321.63667.24. 14615388

[71] P. Sucosky , K. Balachandran , A. Elhammali , H. Jo , and A. P. Yoganathan , “Altered Shear Stress Stimulates Upregulation of Endothelial VCAM‐1 and ICAM‐1 in a BMP‐4– and TGF‐β1–Dependent Pathway,” Arteriosclerosis, Thrombosis, and Vascular Biology 29, no. 2 (2009): 254–260, 10.1161/ATVBAHA.108.176347. 19023092 PMC5467689

[72] E. Kopczyńska and R. Makarewicz , “Endoglin—A Marker of Vascular Endothelial Cell Proliferation in Cancer,” Contemporary Oncology/Współczesna Onkologia 16, no. 1 (2012): 68–71. 23788858 10.5114/wo.2012.27340 PMC3687377

[73] S. E. Duff , C. Li , J. M. Garland , and S. Kumar , “CD105 is Important for Angiogenesis: Evidence and Potential Applications,” The FASEB Journal 17, no. 9 (2003): 984–992, 10.1096/fj.02-0634rev. 12773481

[74] N. Kawelke , M. Vasel , C. Sens , A. Au , S. Dooley , and I. A. Nakchbandi , “Fibronectin Protects from Excessive Liver Fibrosis by Modulating the Availability of and Responsiveness of Stellate Cells to Active TGF‐β,” PLoS ONE 6, no. 11 (2011): 28181, 10.1371/journal.pone.0028181. PMC3225392 22140539

[75] X. Y. Liu , R. X. Liu , F. Hou , et al., “Fibronectin Expression Is Critical for Liver Fibrogenesis In Vivo and In Vitro,” Molecular Medicine Reports 14, no. 4 (2016): 3669–3675, 10.3892/mmr.2016.5673. 27572112 PMC5042748

[76] R. S. Aziz‐Seible and C. A. Casey , “Fibronectin: Functional Character and Role in Alcoholic Liver Disease,” World Journal of Gastroenterology 17, no. 20 (2011): 2482–2499, 10.3748/wjg.v17.i20.2482. 21633653 PMC3103806

[77] R. Bataller and D. A. Brenner , “Liver Fibrosis,” Journal of Clinical Investigation 115, no. 2 (2005): 209–218, 10.1172/JCI24282. 15690074 PMC546435

[78] T. Guo , C. Wantono , Y. Tan , F. Deng , T. Duan , and D. Liu , “Regulators, Functions, and Mechanotransduction Pathways of Matrix Stiffness in Hepatic Disease,” Frontiers in Physiology 14 (2023): 1098129. 36711017 10.3389/fphys.2023.1098129 PMC9878334

[79] C. Ortiz , R. Schierwagen , L. Schaefer , S. Klein , X. Trepat , and J. Trebicka , “Extracellular Matrix Remodeling in Chronic Liver Disease,” Current Tissue Microenvironment Reports 2, no. 3 (2021): 41–52, 10.1007/s43152-021-00030-3. 34337431 PMC8300084

[80] E. Lafoz , M. Ruart , A. Anton , A. Oncins , and V. Hernández‐Gea , “The Endothelium as a Driver of Liver Fibrosis and Regeneration,” Cells 9, no. 4 (2020): 929, 10.3390/cells9040929. 32290100 PMC7226820

[81] M. J. McConnell , E. Kostallari , S. H. Ibrahim , and Y. Iwakiri , “The Evolving Role of Liver Sinusoidal Endothelial Cells in Liver Health and Disease,” Hepatology 78, no. 2 (2023): 649–669, 10.1097/HEP.0000000000000207. 36626620 PMC10315420

[82] E. Wisse , F. Braet , D. Luo , et al., “Structure and Function of Sinusoidal Lining Cells in the Liver,” Toxicologic Pathology 24, no. 1 (1996): 100–111, 10.1177/019262339602400114. 8839287

[83] Y. ZiYue , L. Xin , H. Ying , and C. LiNa , “Role of Liver Sinusoidal Endothelial Cells in Liver Regeneration and the Development of Liver Fibrosis,” Journal of Clinical Hepatology 35, no. 9 (2019): 2072.

[84] R. H. Wenger , D. P. Stiehl , and G. Camenisch , “Integration of Oxygen Signaling at the Consensus HRE,” Science's STKE 2005, no. 306 (2005): re12, 10.1126/stke.3062005re12. 16234508

[85] G. L. Semenza , “Hypoxia‐Inducible Factors in Physiology and Medicine,” Cell 148, no. 3 (2012): 399–408. 22304911 10.1016/j.cell.2012.01.021 PMC3437543

[86] R. H. Wenger , A. Rolfs , H. H. Marti , C. Bauer , and M. Gassmann , “Hypoxia, a Novel Inducer of Acute Phase Gene Expression in a human Hepatoma Cell Line,” Journal of Biological Chemistry 270, no. 46 (1995): 27865–27870, 10.1074/jbc.270.46.27865. 7499259

[87] B. Nath and G. Szabo , “Hypoxia and Hypoxia Inducible Factors: Diverse Roles in Liver Diseases,” Hepatology 55, no. 2 (2012): 622–633. 22120903 10.1002/hep.25497 PMC3417333

[88] A. G. Lai , D. Forde , W. H. Chang , et al., “Glucose and Glutamine Availability Regulate HepG2 Transcriptional Responses to Low Oxygen,” Wellcome Open Research 3 (2018): 126, 10.12688/wellcomeopenres.14839.1. 30345392 PMC6178907

[89] A. Z. Ayob and T. S. Ramasamy , “Prolonged Hypoxia Switched on Cancer Stem Cell‐Like Plasticity in HepG2 Tumourspheres Cultured in Serum‐free media,” In Vitro Cellular & Developmental Biology—Animal 57, no. 9 (2021): 896–911, 10.1007/s11626-021-00625-y. 34750738

[90] M. Minoves , F. Hazane‐Puch , G. Moriondo , et al., “Differential Impact of Intermittent vs. Sustained Hypoxia on HIF‐1, VEGF and Proliferation of HepG2 Cells,” International Journal of Molecular Sciences 24, no. 8 (2023): 6875, 10.3390/ijms24086875. 37108039 PMC10139223

[91] M. K. Rana , J. Srivastava , M. Yang , C. S. Chen , and D. L. Barber , “Hypoxia Increases the Abundance but Not the Assembly of Extracellular Fibronectin during Epithelial Cell Transdifferentiation,” Journal of Cell Science 128, no. 6 (2015): 1083–1089. 25616899 10.1242/jcs.155036 PMC4359919

[92] T. Kietzmann , “Metabolic Zonation of the Liver: the Oxygen Gradient Revisited,” Redox Biology 11 (2017): 622–630, 10.1016/j.redox.2017.01.012. 28126520 PMC5257182

[93] E. Altrock , C. Sens , C. Wuerfel , et al., “Inhibition of Fibronectin Deposition Improves Experimental Liver Fibrosis,” Journal of Hepatology 62, no. 3 (2015): 625–633, 10.1016/j.jhep.2014.06.010. 24946284

[94] H. Yokomori , K. Yoshimura , S. Ohshima , et al., “The Endothelin‐1 Receptor‐Mediated Pathway Is Not Involved in the Endothelin‐1‐Induced Defenestration of Liver Sinusoidal Endothelial Cells,” Liver International 26, no. 10 (2006): 1268–1276, 10.1111/j.1478-3231.2006.01365.x. 17105593

[95] M. Ortega‐Ribera , A. Gibert‐Ramos , L. Abad‐Jordà , et al., “Increased Sinusoidal Pressure Impairs Liver Endothelial Mechanosensing, Uncovering Novel Biomarkers of Portal Hypertension,” JHEP Reports 5, no. 6 (2023): 100722, 10.1016/j.jhepr.2023.100722. 37151732 PMC10154975

[96] J. Zhou , J. Wang , L. Zhang , C. Zhang , and C. Tian , “Defenestration of Liver Sinusoidal Endothelial Cells: the Trigger of Liver Fibrosis,” Pharmaceuticals 18, no. 6 (2025): 893, 10.3390/ph18060893. 40573288 PMC12196518

[97] J. X. Zhang , W. Pegoli Jr. , and M. G. Clemens , “Endothelin‐1 Induces Direct Constriction of Hepatic Sinusoids,” American Journal of Physiology 266, no. 4 Pt 1 (1994): G624–G632. 8179001 10.1152/ajpgi.1994.266.4.G624

[98] Y. Iwakiri , “Endothelial Dysfunction in the Regulation of Cirrhosis and Portal Hypertension,” Liver International 32, no. 2 (2012): 199–213, 10.1111/j.1478-3231.2011.02579.x. 21745318 PMC3676636

[99] A. K. Khimji and D. C. Rockey , “Endothelin and Hepatic Wound Healing,” Pharmacological Research 63, no. 6 (2011): 512–518, 10.1016/j.phrs.2011.03.005. 21421048 PMC4129451

[100] A. Nesci , V. Ruggieri , V. Manilla , et al., “Endothelial Dysfunction and Liver Cirrhosis: Unraveling of a Complex Relationship,” International Journal of Molecular Sciences 25, no. 23 (2024): 12859, 10.3390/ijms252312859. 39684569 PMC11640898

[101] N. S. Bhise , J. Ribas , V. Manoharan , et al., “Organ‐on‐a‐chip Platforms for Studying Drug Delivery Systems,” Journal of Controlled Release 190 (2014): 82–93, 10.1016/j.jconrel.2014.05.004. 24818770 PMC4142092

[102] B. R. Ware and S. R. Khetani , “Engineered Liver Platforms for Different Phases of Drug Development,” Trends in Biotechnology 35, no. 2 (2017): 172–183, 10.1016/j.tibtech.2016.08.001. 27592803 PMC5253249

[103] J. Y. Chiang , “Bile Acid Metabolism and Signaling,” Comprehensive Physiology 3, no. 3 (2013): 1191–1212, 10.1002/j.2040-4603.2013.tb00517.x. 23897684 PMC4422175

[104] Y. Moon , B. Park , and H. Park , “Hypoxic Repression of CYP7A1 through a HIF‐1α‐ and SHP‐Independent Mechanism,” BMB Reports 49, no. 3 (2016): 173–178, 10.5483/BMBRep.2016.49.3.188. 26521940 PMC4915232

[105] C. Zhang , Y. Zhao , M. Yu , J. Qin , B. Ye , and Q. Wang , “Mitochondrial Dysfunction and Chronic Liver Disease,” Current Issues in Molecular Biology 44, no. 7 (2022): 3156–3165. 35877442 10.3390/cimb44070218 PMC9319137

[106] P. Banerjee , N. Gaddam , V. Chandler , and S. Chakraborty , “Oxidative Stress–Induced Liver Damage and Remodeling of the Liver Vasculature,” The American Journal of Pathology 193, no. 10 (2023): 1400–1414. 37355037 10.1016/j.ajpath.2023.06.002

[107] S. Hu , S. Liu , Y. Bian , et al., “Single‐cell Spatial Transcriptomics Reveals a Dynamic Control of Metabolic Zonation and Liver Regeneration by Endothelial Cell Wnt2 and Wnt9b,” Cell Reports Medicine 3, no. 10 (2022): 100754, 10.1016/j.xcrm.2022.100754. 36220068 PMC9588996

[108] T. T. Gordon‐Walker , K. Bove , and G. Veldtman , “Fontan‐associated Liver Disease: a Review,” Journal of Cardiology 74, no. 3 (2019): 223–232, 10.1016/j.jjcc.2019.02.016. 30928109

[109] T. Diamond and N. Ovchinsky , “Fontan‐Associated Liver Disease: Monitoring Progression of Liver Fibrosis,” Clinical Liver Disease 11, no. 1 (2018): 1–5, 10.1002/cld.681. 30992779 PMC6385938

[110] S. Dooley and P. ten Dijke , “TGF‐β in Progression of Liver Disease,” Cell and Tissue Research 347, no. 1 (2012): 245–256, 10.1007/s00441-011-1246-y. 22006249 PMC3250614

[111] I. Fabregat , J. Moreno‐Càceres , A. Sánchez , et al., “TGF ‐β Signalling and Liver Disease,” The FEBS Journal 283, no. 12 (2016): 2219–2232, 10.1111/febs.13665. 26807763

[112] F. Xu , C. Liu , D. Zhou , and L. Zhang , “TGF‐β/SMAD Pathway and Its Regulation in Hepatic Fibrosis,” Journal of Histochemistry & Cytochemistry 64, no. 3 (2016): 157–167, 10.1369/0022155415627681. 26747705 PMC4810800

[113] S. Shetty , P. F. Lalor , and D. H. Adams , “Liver Sinusoidal Endothelial Cells — Gatekeepers of Hepatic Immunity,” Nature Reviews Gastroenterology & Hepatology 15, no. 9 (2018): 555–567, 10.1038/s41575-018-0020-y. 29844586 PMC7096836

[114] P. A. Knolle and D. Wohlleber , “Immunological Functions of Liver Sinusoidal Endothelial Cells,” Cellular & Molecular Immunology 13, no. 3 (2016): 347–353, 10.1038/cmi.2016.5. 27041636 PMC4856811

[115] A. Baiocchini , F. Del Nonno , C. Taibi , et al., “Liver Sinusoidal Endothelial Cells (LSECs) Modifications in Patients with Chronic hepatitis C,” Scientific Reports 9, no. 1 (2019): 8760, 10.1038/s41598-019-45114-1. 31217430 PMC6584733

[116] L. Ning , S. Zanella , M. L. Tomov , et al., “Targeted Rapamycin Delivery via Magnetic Nanoparticles to Address Stenosis in a 3D Bioprinted in Vitro Model of Pulmonary Veins,” Advanced Science 11, no. 26 (2024): 2400476. 38696618 10.1002/advs.202400476 PMC11234432

[117] B. Hwang , L. Korsnick , M. Shen , et al., “FSTL‐1 Loaded 3D Bioprinted Vascular Patch Regenerates the Ischemic Heart Tissue,” iScience 27, no. 10 (2024): 110770. 39398249 10.1016/j.isci.2024.110770 PMC11466656

[118] C. J. Gil , C. J. Evans , L. Li , et al., “Leveraging 3d Bioprinting and Photon‐Counting Computed Tomography to Enable Noninvasive Quantitative Tracking of Multifunctional Tissue Engineered Constructs,” Advanced healthcare materials 12 (2023): 2302271. 10.1002/adhm.202302271 PMC10842604 37709282

[119] M. L. Tomov , A. Cetnar , K. Do , H. Bauser‐Heaton , and V. Serpooshan , “Patient‐Specific 3‐Dimensional–Bioprinted Model for in Vitro Analysis and Treatment Planning of Pulmonary Artery Atresia in Tetralogy of Fallot and Major Aortopulmonary Collateral Arteries,” Journal of the American Heart Association 8, no. 24 (2019): 014490, 10.1161/JAHA.119.014490. PMC6951056 31818221

[120] V. Serpooshan , T. M. Quinn , N. Muja , and S. N. Nazhat , “Characterization and Modelling of a Dense Lamella Formed during Self‐compression of Fibrillar Collagen Gels: Implications for Biomimetic Scaffolds,” Soft Matter 7, no. 6 (2011): 2918–2926, 10.1039/c0sm00691b.

[121] V. Serpooshan , N. Muja , B. Marelli , and S. N. Nazhat , “Fibroblast Contractility and Growth in Plastic Compressed Collagen Gel Scaffolds with Microstructures Correlated with Hydraulic Permeability,” Journal of Biomedical Materials Research Part A 96A, no. 4 (2011): 609–620, 10.1002/jbm.a.33008. 21268235

[122] M. L. Tomov , L. Perez , L. Ning , et al., “A 3D Bioprinted in Vitro Model of Pulmonary Artery Atresia to Evaluate Endothelial Cell Response to Microenvironment,” Advanced Healthcare Materials 10 (2021): 2100968, 10.1002/adhm.202100968. PMC8823098 34369107

[123] A. D. Cetnar , M. L. Tomov , L. Ning , et al., “Patient‐Specific 3D Bioprinted Models of Developing Human Heart,” Advanced Healthcare Materials 10, no. 15 (2021): 2001169. 10.1002/adhm.202001169 PMC8175477 33274834

[124] M. Shokouhimehr , A. S. Theus , A. Kamalakar , et al., “3D Bioprinted Bacteriostatic Hyperelastic Bone Scaffold for Damage‐Specific Bone Regeneration,” Polymers 13, no. 7 (2021): 1099, 10.3390/polym13071099. 33808295 PMC8036866

[125] W. C. Oliver and G. M. Pharr , “An Improved Technique for Determining Hardness and Elastic Modulus Using Load and Displacement Sensing Indentation Experiments,” Journal of Materials Research 7, no. 6 (1992): 1564–1583, 10.1557/JMR.1992.1564.

[126] V. Serpooshan and M. Guvendiren , “Editorial for the Special Issue on 3D Printing for Tissue Engineering and Regenerative Medicine,” Micromachines 11 (2020): 366. 32244506 10.3390/mi11040366 PMC7230784

[127] J. Westerweel and F. Scarano , “Universal Outlier Detection for PIV Data,” Experiments in Fluids 39, no. 6 (2005): 1096–1100, 10.1007/s00348-005-0016-6.

