# Supplementary material for: A Dynamic 3D Human Liver Sinusoid Model for Mechanistic Interrogation of Fontan‐Associated Liver Disease
Source: Adv Sci (Weinh). 2026 Apr 8;13(30):e24337. doi: 10.1002/advs.202524337 (PMC13248770; doi:10.1002/advs.202524337)
Supplement: Supplementary file 1 — Supporting File: advs74851‐sup‐0001‐SuppMat.docx. [file ADVS-13-e24337-s001.docx]

**Supporting Information**


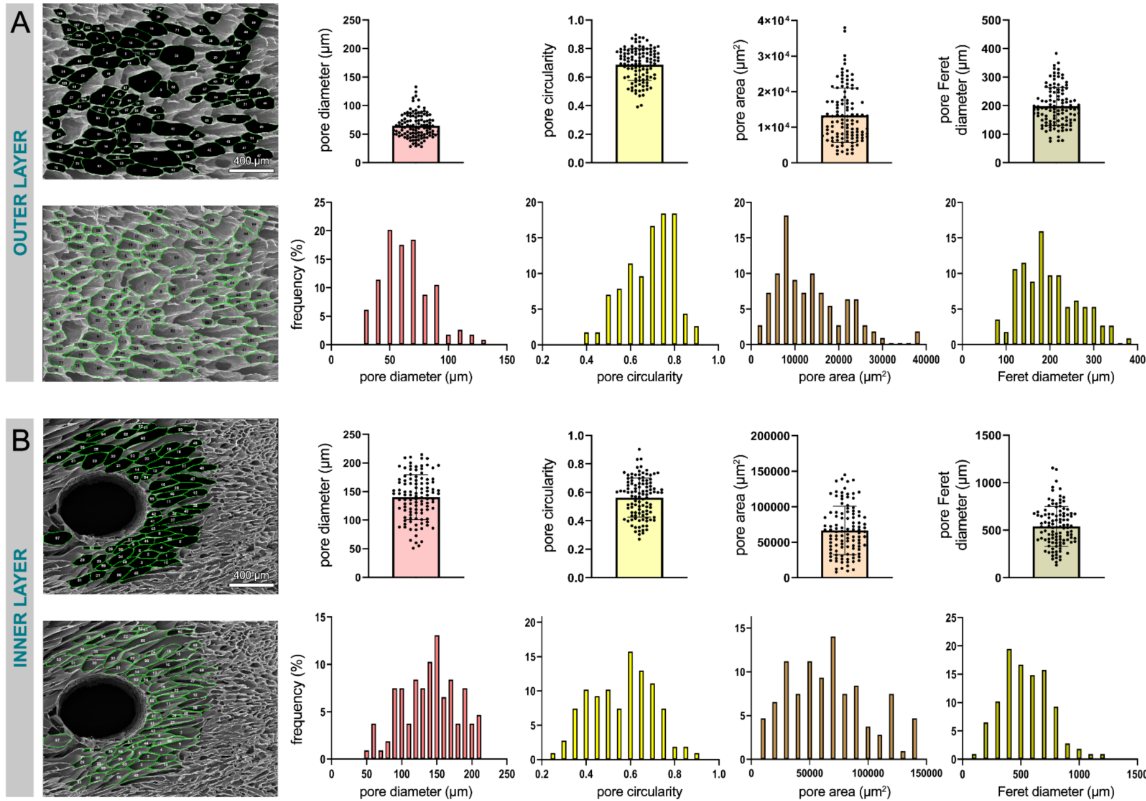


**Supplementary Figure S1. Scanning electron microscopy (SEM) characterization of the 3D engineered models of hepatic sinusoids.** **A-B:** The image analysis workflow conducted on the SEM images (left) and the analysis results (right) are presented for the outer layer (**A**) and inner layer (**B**) of constructs. Pore structure analysis results are presented for pore diameter, circularity, surface area, and Feret diameter (*n* = 4 per group).


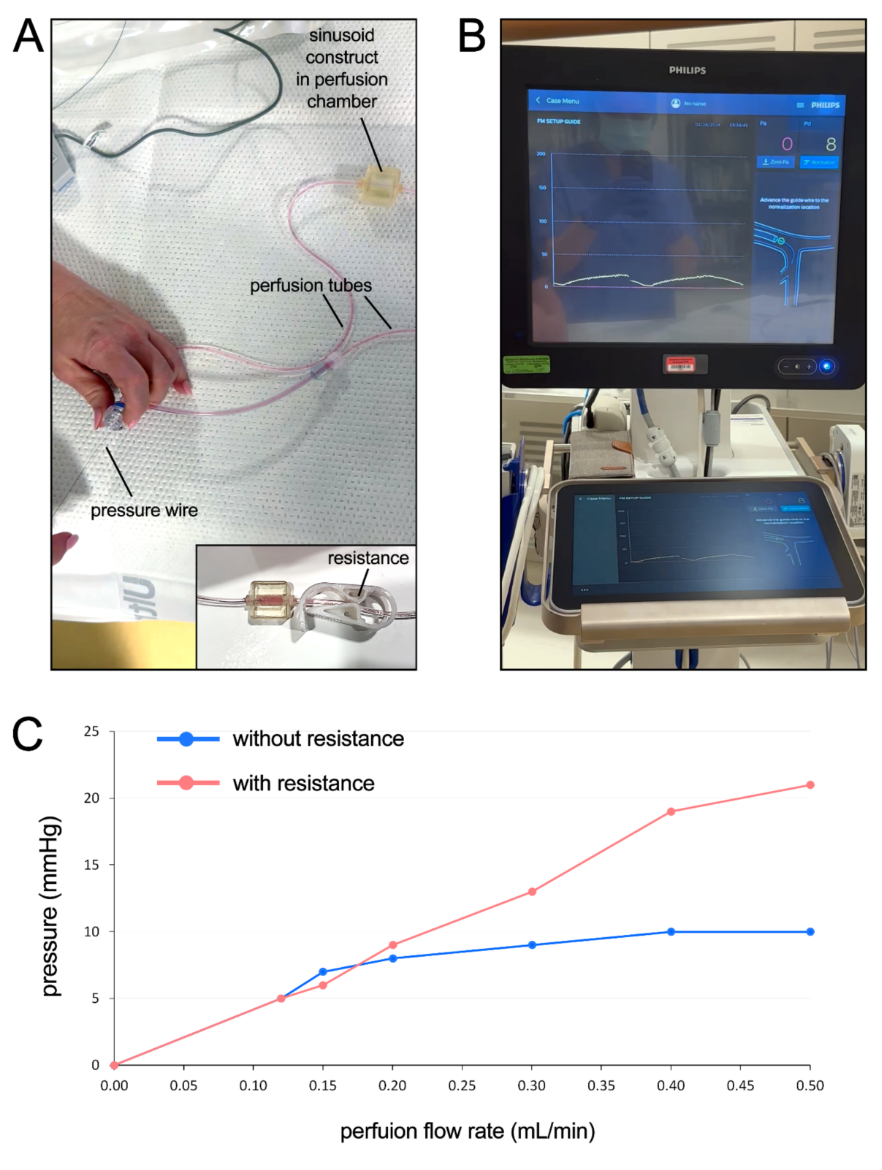


**Supplementary Figure S2. Measurement of intraluminal pressure in hepatic sinusoid constructs.** **A-B:** The experimental set-up used in the catheterization lab to measure pressure in the mid-channel space. **C:** Measured pressure values for varying flow rates perfused through the bioengineered sinusoid constructs. Results are presented for perfusion without and with downstream resistance to simulate venous outflow obstruction in the Fontan associated liver disease.


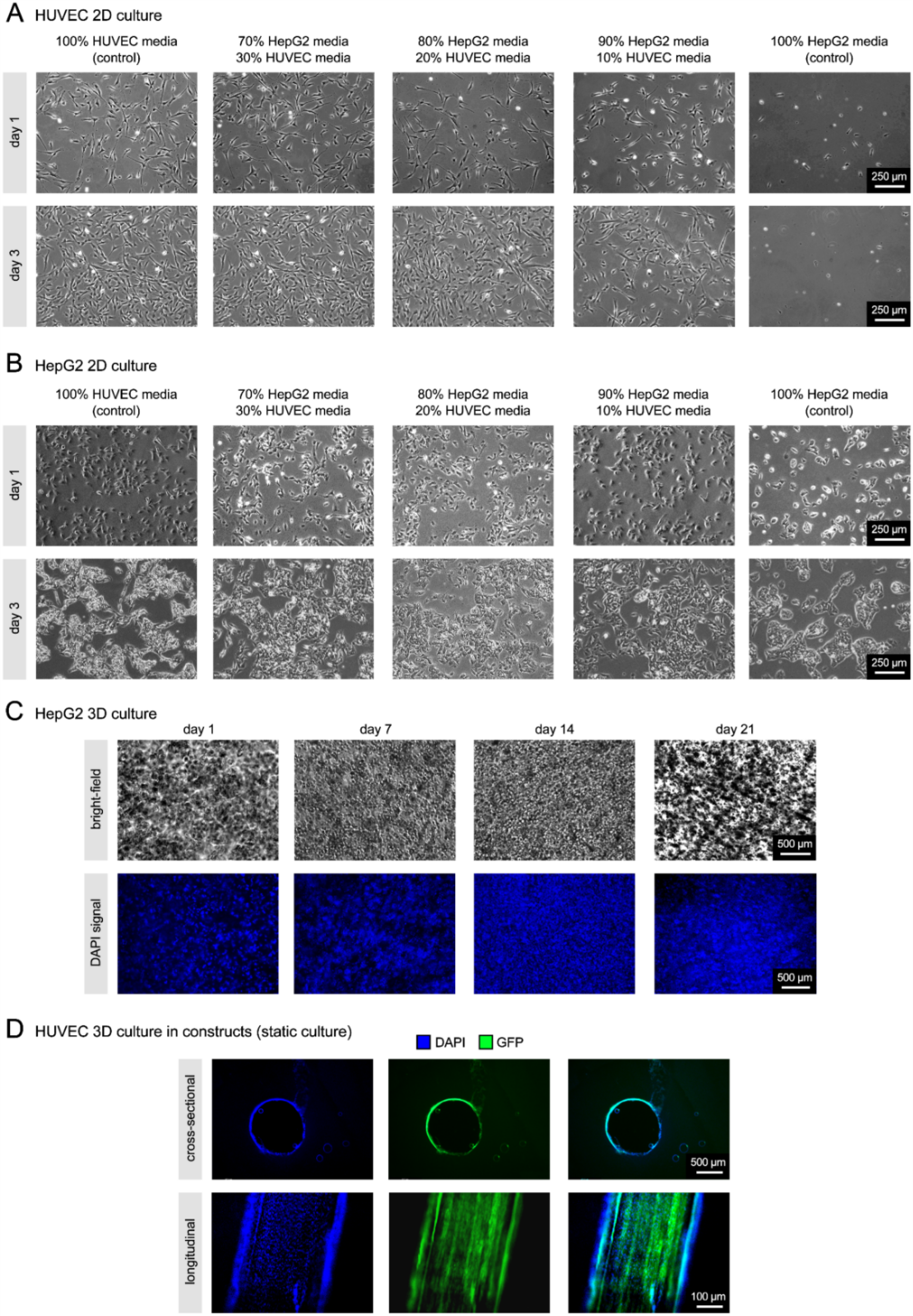


**Supplementary Figure S3. Immunostaining characterization of human umbilical vein endothelial cells (HUVECs) and hepatocytes (HepG2) in 2D and 3D culture.** **A-B:** 2D culture assays of HUVECs (**A**) and HepG2 cells (**B**) using various culture media ratios to determine the optimal media composition. **C:** HepG2 culture in 3D scaffold over 21 days of culture. **D:** 3D static (no flow, 14 days) culture of engineered constructs seeded with HUVECs.


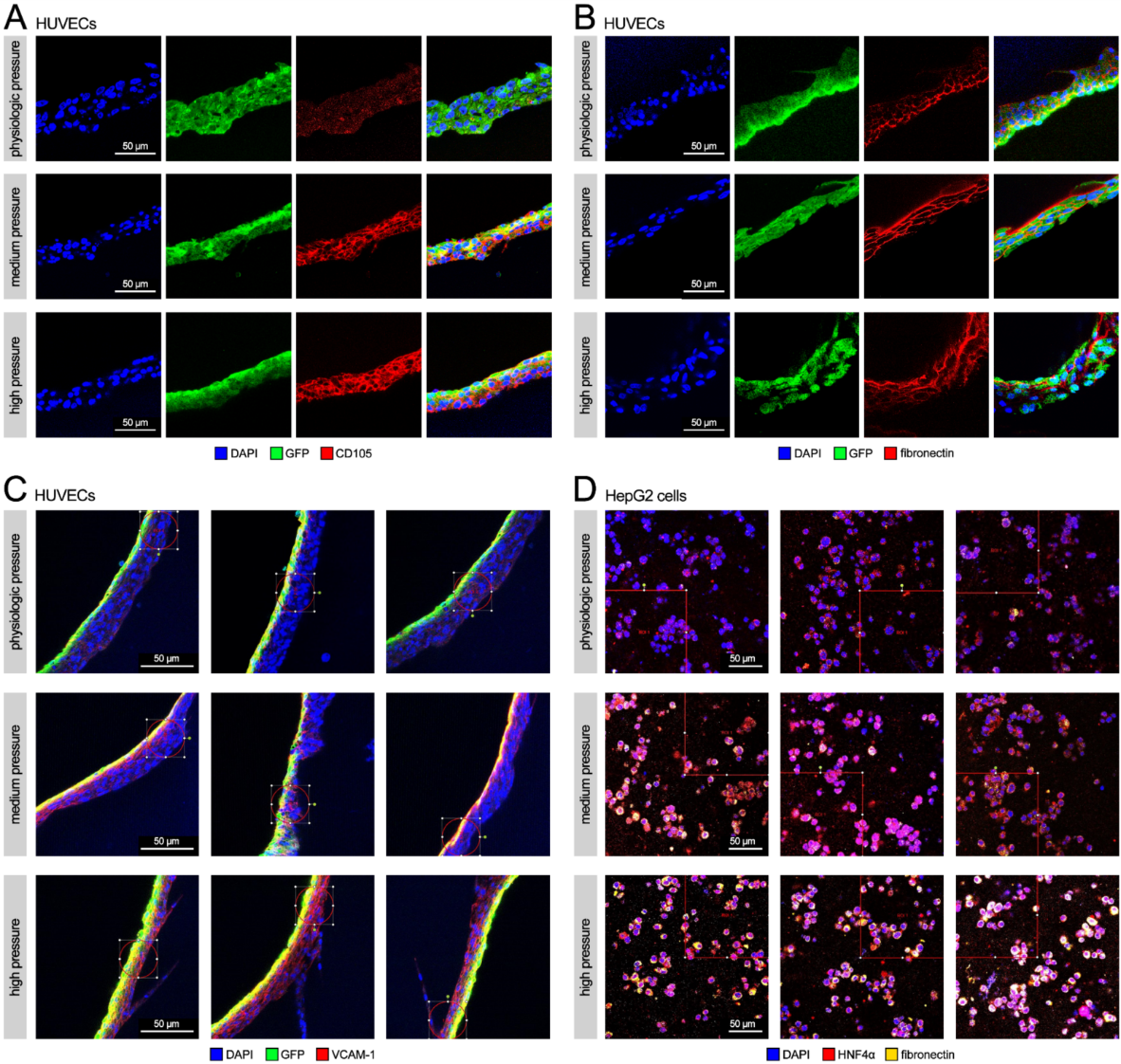


**Supplementary Figure Ssup-4. Immunostaining analysis of cell growth and function in 3D constructs under varying pressure conditions in dynamic culture.** **A-C:** Imaging of human umbilical vein endothelial cells (HUVECs), forming a thin layer on the surface of channel lumens in the sinusoid constructs. Three culture groups include: physiological, medium, and high pressure. **D:** Immunostaining of human hepatocytes (HepG2 cells) within the bulk (outer layer) of 3D constructs.
